# Readjoiner: a fast and memory efficient string graph-based sequence assembler

**DOI:** 10.1186/1471-2105-13-82

**Published:** 2012-05-06

**Authors:** Giorgio Gonnella, Stefan Kurtz

**Affiliations:** 1 Center for Bioinformatics, University of Hamburg, Bundesstrasse 43, 20146, Hamburg, Germany

## Abstract

**Background:**

Ongoing improvements in throughput of the next-generation sequencing technologies challenge the current generation of de novo sequence assemblers. Most recent sequence assemblers are based on the construction of a de Bruijn graph. An alternative framework of growing interest is the assembly string graph, not necessitating a division of the reads into *k*-mers, but requiring fast algorithms for the computation of suffix-prefix matches among all pairs of reads.

**Results:**

Here we present efficient methods for the construction of a string graph from a set of sequencing reads. Our approach employs suffix sorting and scanning methods to compute suffix-prefix matches. Transitive edges are recognized and eliminated early in the process and the graph is efficiently constructed including irreducible edges only.

**Conclusions:**

Our suffix-prefix match determination and string graph construction algorithms have been implemented in the software package Readjoiner. Comparison with existing string graph-based assemblers shows that Readjoiner is faster and more space efficient. Readjoiner is available at http://www.zbh.uni-hamburg.de/readjoiner.

## Background

The *de novo* sequence assembly problem is to reconstruct a target sequence from a set of sequence reads. The classical approach to *de novo* assembly consists of three phases: overlap, layout and consensus. During the overlap phase, suffix-prefix matches among all pairs of sequence reads are computed, and turned into an overlap graph [1]. In the layout phase the location of the reads with respect to each other is determined. In the consensus phase the target sequence is reconstructed, by selecting a base for each position.

The introduction of the massively parallel next-generation DNA sequencing technologies has led to a considerable increase in the amount of data typically generated by sequencing experiments. For example, as of January 2012, the HiSeq2000 sequencer of Illumina delivers sets of 100 bp reads with a total length of up to 600 Gbp [2]. Sequence analysis software tools developed only a few years ago are often unable to deal with such large amounts of short reads: This has led to a gap between the ability to produce sequence data and the capability to assemble and analyze them [3].

The computation of the overlap graph is the most time and space consuming of the three phases, and was considered a bottleneck in the computation. Therefore, alternative methods were developed avoiding an explicit overlap computation. An approach which proved to be effective is based on the enumeration of all *k*-mers of the reads and their representation in a de Bruijn graph, as first proposed by [4]. This concept is applied in several popular short reads assemblers such as Velvet [5], EULER-SR [6] and Abyss [7].

The de Bruijn graph describing the *k*-mer spectrum of the read set has interesting properties for the solution of the assembly problem, such as the collapsing of different instances of sequence repeats into common paths of the graph. However, reducing short reads into even shorter units compromises the ability of disambiguation of short repeats. Myers [8] presented an alternative framework, the assembly string graph. Like in the de Bruijn graph, repeats still collapse into common graph elements. There are two main advantages of the string graph, compared to the de Bruijn graph: At first, it does not require to split the reads into *k*-mers. Secondly, a string graph always retains read coherence, i.e. each path in the string graph represents a valid assembly of the reads.

Edena [9] was the first available implementation of a string graph-based assembler. It computes suffix-prefix matches using a suffix array [10] representing all suffixes of the reads. From these, the complete overlap graph is constructed before transitive edges are removed using an algorithm described in [8].

A more space-efficient approach to the string graph construction has been presented in [11] and was implemented in the open source String Graph Assembler (SGA) [12]. SGA computes suffix-prefix matches using an algorithm based on the Burrows and Wheeler transform (BWT), allowing to classify suffix-prefix matches as transitive or irreducible, so that the string graph can directly be constructed.

Recently, a compact representation for exact-match overlap graphs has been described in [13], together with a fast construction algorithm, which has been implemented in the string graph-based assembler LEAP.

In this paper, we present new efficient algorithms for the computation of irreducible suffix-prefix matches and the construction of the assembly string graph. These are implemented in a new string graph based sequence assembler *Readjoiner*. To validate our approach, we compared *Readjoiner* with the current implementations of Edena, LEAP and SGA. *Readjoiner* proved to be considerably faster than previous competitors, or uses less memory. In fact, *Readjoiner* is able to handle very large datasets using limited resources: for example, a short reads dataset consisting of 115 Gbp could be assembled on a single core in 51 hours using 52 GB RAM.

All string graph-based assemblers aim at constructing the same graph: However, the algorithms and data structures employed in Edena, LEAP, SGA and *Readjoiner* differ considerably. LEAP employs a compact representation of the overlap graph, while *Readjoiner* circumvents the construction of the full overlap graph. Both Edena and SGA are based on explicit index structures (suffix array and FM-index, respectively) representing all suffixes of all reads in the read set, while *Readjoiner* enumerates and sorts only a proper subset of the suffixes of the reads, and efficiently inserts them into buckets, which can be processed independently from each other.

## Methods

### Basic definitions

Let *w* be a string of length *n* of symbols over an alphabet Σ. *w*[*i*] denotes the *i*th symbol of *w* and *w*[*i*…*j*] the substring of *w* from position *i* to *j*, 1 ≤ *i*, *j* ≤ *n*. *w*[1…*i*] is the *prefix* of *w* ending at position *i* and *w*[*j*…*n*] is the *suffix* of *w* starting at position *j*. A substring of *w* is *proper* if it is different from *w*. A substring of *w* is *internal* if it is neither a prefix nor a suffix of *w*.

A read *r* is a string over the alphabet {*A*, *C*, *G*, *T*} which is assumed to be sorted by the alphabetical order < such that *A* < *C* < *G* < *T*. ⊴ denotes the lexicographic order of all substrings of the reads induced by the alphabetical order < . Let *n* be the length of *r*. The reverse complement of *r*, denoted by $\overset{―}{r}$, is the sequence $\overset{―}{r[n]}\ldots \overset{―}{r[1]}$, where $\overset{―}{a}$ indicates the Watson-Crick complement of base *a*.

### Computing suffix- and prefix-free read sets

The first step of our approach for assembling a collection of reads is to eliminate reads that are prefixes or suffixes of other reads. Here we describe a method to recognize these reads. Consider an ordered set $R=(r_{1},\ldots ,r_{m})$ of reads, possibly of variable length, in which some reads may occur more than once (so R is indeed a multiset). We assume that, for all *i*, 1 ≤ *i* ≤ *m*, the *i*th read *r*_*i*_ in R is virtually padded by a *sentinel symbol* $_*i*_ at the right end and that the alphabetical order < is extended such that *A* < *C* < *G* < *T* < $_1_ < $_2_ < · · · < $_*m*_.

We define a binary relation ≺ on R such that *r*_*i*_ ≺ *r*_*j*_ if and only if *i* < *j*. That is, ≺ reflects the order of the reads in the input. R is *prefix-free* if for all reads *r* in R there is no *r*′ in R∖{r} such that *r* is a prefix of *r*′. R is *suffix-free* if for all *r* in R there is no read *r*′ in R∖{r} such that *r* is a suffix of *r*′.

To obtain a prefix- and suffix-free set of reads we lexicographically sort all reads using a modified radixsort for strings, as described in [14]. In this algorithm, the strings to be sorted are first inserted into buckets according to their first character. Each bucket is then sorted recursively according to the next character of all reads in the bucket. A bucket always consists of reads which have a common prefix. Once a bucket is smaller than some constant, the remaining suffixes of the reads in the bucket are sorted by insertion sort [15].

During the sorting process, the length of the longest common prefix (lcp) of two lexicographically consecutive reads is calculated as a byproduct. For two lexicographically consecutive reads *r* and *r*′ with an lcp of length ℓ = |*r*|, we can conclude that *r* is a prefix of *r*′. If ℓ < |*r*′|, then *r* is a proper prefix of *r*′ and we mark *r*. If ℓ = |*r*′|, then *r* and *r*′ are identical and we mark the read which is larger according to the binary relation ≺ .

To handle reverse complements and to mark reads which are suffixes of other reads, one simply applies this method to the multiset $\overset{―}{R}=(r_{1},\ldots ,r_{m},r_{m+1},\ldots ,r_{2m})$ where $r_{m+i}=\bar{r_{i}}$ for all *i*, 1 ≤ *i* ≤ *m*. As $\overset{―}{R}$ includes the reverse complements of the reads, the method also marks reads which are suffixes of other reads. This is due to the observation that if read *r* is a suffix of read *r*′, then $\overset{―}{r}$ is a prefix of $\overset{―}{r^{\prime }}$.

In a final step of the algorithm one eliminates all reads from R which have been marked. The remaining unmarked reads from R are processed further. The algorithm to compute a suffix- and prefix-free set of reads runs in $O(m\frac{\lambda_{\text{max}}}{\omega })$ time, where *λ*_max_ is the maximum length of a read and *ω* is the machine’s word size. As we consider *λ*_max_ to be a constant (which does not imply that the reads are all of the same length), the algorithm runs in *O*(*m*) time.

### Computing suffix-prefix matches

Suppose that R is a suffix- and prefix-free set of *m* reads. Let ℓ_*min*_ > 0 be the minimum length parameter. The set SPM(R) of *suffix-prefix matches* (*SPM*s, for short) is the smallest set of triples 〈*r*, *r*′, ℓ〉 such that r,r′∈R and strings *u*, *v*, *w* exist such that *r* = *uv*, *r*′ = *vw*, and |v| =ℓ ≥ ℓ_*min*_. ℓ is the *length* of a suffix-prefix match 〈*r*, *r*′, ℓ〉 . The *suffix-prefix matching problem* is to find all suffix-prefix matches. As the reads of length smaller than ℓ_*min*_ cannot, by definition, contribute any *SPM*, we can ignore them and thus we assume that R only contains reads of length at least ℓ_*min*_.

The method to solve the *suffix-prefix matching problem* presented here consists of two main algorithms. The first algorithm identifies and lexicographically sorts all SPM-relevant suffixes, *i.e.* a subset of all suffixes of all reads from which one can compute all suffix-prefix matches. The second algorithm enumerates these matches given the sorted list of all SPM-relevant suffixes.

Consider a suffix-prefix match 〈*r*, *r*′, ℓ〉 . By definition, the suffix of length ℓ of *r* exactly matches the prefix of length ℓ of *r*′. Obviously, the suffix of *r* involved in the match starts at some position *j*, $2\leq j\leq |r|-\ell_{\min}+1$ in *r*. This implies that *r* must be at least of length ℓ_*min*_ + 1. The suffix cannot start at the first position in *r*, as otherwise *r* would be a prefix of some other read, contradicting our assumption that R is prefix-free.

To enumerate the set of all suffix-prefix matches of length at least ℓ_*min*_, we preprocess all reads and determine all proper suffixes of the reads which may be involved in a suffix-prefix match. More precisely, for all reads *r* we determine all *matching candidates*, i.e. all proper suffixes *s* of *r* such that the length of *s* is at least ℓ_*min*_ and there is a read *r*′ such that *s* and *r*′ have a common prefix of length at least *k*, where *k* is an user-defined parameter satisfying $k\leq min\{\ell_{\min},\frac{\omega }{2}\}$. There are two reasons for imposing this constraint on *k*: First, we want to represent a string of length *k* over an alphabet of size 4 in one machine word, thus $k\leq \frac{\omega }{2}$. Second, the suffixes of the reads from which we take the prefixes of length *k* have minimum length ℓ_*min*_, thus we choose *k* ≤ ℓ_*min*_.

The set of all matching candidates and all reads forms the *set of all* (ℓ_*min*_, *k*)*-SPM-relevant suffixes*. For simplicity sake, we use the notion *SPM-relevant suffixes* if ℓ_*min*_ and *k* are clear from the context. While all *SPM*s can be constructed from the SPM-relevant suffixes, not all SPM-relevant suffixes lead to an *SPM*.

#### An efficient algorithm for identifying and sorting all SPM-relevant suffixes

The first two phases of our algorithm follow a strategy that is borrowed from the counting sort algorithm [15]. Like this, our algorithm has a counting phase and an insertion phase. However, in our problem, the elements (i.e. SPM-relevant suffixes) to be sorted are only determined during the algorithm. Moreover, the number of keys (i.e. initial *k*-mers) whose occurrences are counted is on the order of the number of elements to be sorted. Therefore, in a space efficient solution, it is not trivial to access a counter given a key. We have developed a time and space efficient method to access the counter for a key, exploiting the fact that counting and inserting the SPM-relevant suffixes does not have to be done immediately. Instead, the items to be counted/inserted are first buffered and then sorted. A linear scan then performs the counting or inserting step.

In contrast to counting sort, our algorithm uses an extra sorting step to obtain the final order of elements pre-sorted in the insertion phase. Under the assumption that the maximum read length is a constant (which does not imply that the reads are all of the same length), our algorithm runs in *O*(*n*) time and space, where *n* is the total length of all reads. To the best of our knowledge a method employing a similar strategy has not yet been developed for the suffix-prefix matching problem.

We first give a description of our algorithm using string notation. In a separate section, we explain how to efficiently implement the algorithm. In the following, we only consider the reads in the forward direction. However, it is not difficult to extend our method to also incorporate the reverse complements of the reads and we comment on this issue at the end of the methods section.

The *initial kmer* of some sequence *s* is the prefix of *s* of length *k*. To determine the matching candidates efficiently, we first enumerate the initial *k*-mers of all reads and store them in a table of size *m*. This can be done in *O*(*m*) time. The notion *table size* always refers to the number of entries in the table. The next step lexicographically sorts the *k*-mers in the table in ascending order. This string sorting problem can be transformed into an integer sorting problem (see Implementation) which can be solved by radixsort [15] in *O*(*m*) time and *O*(*m*) extra working space.

In the next step, a linear scan of the sorted *k*-mers removes duplicates from the table and counts how many times each *k*-mer occurs in the table. This scan requires *O*(*m*) time. Let *d* ≤ *m* be the number of different *k*-mers. These can be stored in a table *K* of size *d*.

The counts for the elements in *K* require another table *C* of size *d*. In addition to the duplicate removal and counting, the linear scan of the sorted *k*-mers constructs two sets *P* and *Q*, the size of which depends on two user defined parameters *k*′ ≤ *k* and *k* ″≤ *k*. *P* is the set of all initial *k*′-mers of the reads. *Q* is the set of all *k*-mers r[k−k′′+1…k] for some r∈R. We assume that elements can be added to *P* and *Q* in constant time and that membership in these sets can be decided in constant time. Thus the linear scan constructs *P* and *Q* in *O*(*m*) time. As *P* is a subset of a set of size $4^{k^{\prime }}$, *P* can be stored in $4^{k^{\prime }}$ bits. *Q* requires $4^{k\prime \prime }$ bits.

Up until now, only the initial *k*-mers of the reads were considered, resulting in a sorted table *K* of *d* non-redundant keys (i.e. initial *k*-mers of reads), a table *C* of size *d* for counting *k*-mers and two sets *P* and *Q*. By construction, each count in *C* is at least 1 and the sum of the counts is *m*. The next task is to enumerate, for all reads *r*, the suffixes of *r* at all positions *j*, $2\leq j\leq |r|-\ell_{\min}+1$. *r* has |*r*| − ℓ_*min*_ such suffixes. For each such suffix *s* (which by construction is of length ≥ ℓ_*min*_), one extracts two strings *v* = *s*[1…*k*′] and w=s[k−k′′+1…k]. If *v* does not occur in *P*, then *v* is not a prefix of any read in R and thus *s* is not a matching candidate and can be discarded. If *w* does not occur in *Q*,  then w≠r[k−k′′+1…k] for all reads r∈R and thus *s* is not a matching candidate and can be discarded. Thus *P* and *Q* serve as filters to efficiently detect suffixes which can be discarded. For read *r* the suffixes *s* and corresponding strings *v* and *w* can be enumerated in *O*(|*r*| − ℓ_*min*_) time. Checking membership in *P* and in *Q* requires constant time. Therefore, each read *r* is processed in *O*(|*r*| − ℓ_*min*_) time. Thus the enumeration and checking requires $O(\sum_{r\in R}|r|-m\;\ell_{\min})$ time altogether.

The next task is to process a suffix, say *s* which has passed the *P*-filter and the *Q*-filter. That is, *v* = *s*[1…*k*′] ∈ *P* and w=s[k−k′′+1…k]∈Q holds. One now has to check if *u* = *s*[1…*k*] occurs in *K* to verify if *s* is a matching candidate. If the latter is true, the appropriate counter needs to be incremented. Hence this is the counting phase of our algorithm. The simplest way to check the occurrence of *u* in *K*, is to perform a binary search, taking *u* as the key. However, this would require *O*(*log*_2_*d*) time for each *k*-mer passing the filters. This is too slow. Using a hash table turned out to be too slow as well and would require too much extra space, which we do not want to afford.

We propose an efficient method that works as follows: Store each *k*-mer *s*[1.*k*] passing the *P* and the *Q*-filter in a buffer *B* of fixed size $b=\frac{d}{\gamma }$ for some constant *γ* > 1. Once *B* is full or all *k*-mers have been added to *B*, sort the elements in *B* in ascending lexicographic order. Then perform a binary search in *K*, but only for the first element in *B*, say *x*. As *B* is sorted, *x* is the smallest element. The binary search for *x* in *K* finds the smallest element in *K* greater than or equal to *x* using *O*(*log*_2 _*d*) time. If such an element occurs in *K*, say at index *e*, then simultaneously scan *B* beginning with the first index and *K* beginning at index *e*. For any element in *B* that is equal to an element in *K*, say at index *i* in *K*, increment the counter in *C* at the same index.

This simultaneous linear scan of *B* and (a part of) *K* takes *O*(*b* + *d*) time and finds all *k*-mers from *B* occurring in *K*. Once the scan and the associated increments are done, the buffer is emptied for the next round. Suppose that there are in total *b*^∗^*k*-mers that have passed *B*. Thus there are $\left\lceil \frac{b^{*}}{b}\right\rceil$ rounds filling the buffer. Each round is associated with a sorting step, a binary search and a linear scan. Sorting requires *O*(*b*) time using radixsort. This gives a running time of $O\left(\frac{b^{*}}{b}(b+log_{2}d+(b+d))\right)=O\left(\frac{b^{*}}{b}(b+d)\right)=O\left(\frac{b^{*}}{b}(b+b\gamma )\right)=O(b^{*}(1+\gamma ))=O(b^{*})$. As $b^{*}\leq n:=\sum_{r\in R}|r|$, the running time is linear in the total length of the reads.

Once all reads have been processed, for any initial *k*-mer *u* of any read, the following holds: If *u* is the *i*th initial *k*-mer in *K*, then *C*[*i*] is the number of SPM-relevant suffixes of which *u* is a prefix. To prepare for the insertion phase, compute the partial sums of *C* in an array *π* of size *d* + 1, such that *π*[0] = *C*[0], π[i]=π[i−1]+C[i] for all *i*, 1 ≤ *i* ≤ *d* − 1, and *π*[*d*] = *π*[*d* − 1]. *π*[*d*] is the number of all SPM-relevant suffixes. One creates a table *S* of size *g*: = *π*[*d*] to hold pairs of read numbers and read offsets. As in the counting phase, enumerate all suffixes of reads of length at least ℓ_*min*_ passing the *P*- and the *Q*-filter. Suppose that *s* is such a suffix of read number *p* and with read offset *q*. Let *u* be the initial *k*-mer of *s*. Then we store (*p*, *q*, *u*) in a buffer *B*′ of fixed size $\frac{b}{2}$. We choose this buffer size, as the elements in *B*′ require twice as much space as the elements in *B*. As in the counting phase, sort the buffer in lexicographic order of the *k*-mers it stores, and then process the buffer elements using the *k*-mer, say *u*, as a key to determine if *u* matches some element in *K*, say at index *i*. Then insert (*p*, *q*) at index *π*[*i*] − 1 in *S* and decrement *π*[*i*].

After all *b*^∗^ elements passed the buffer and have been processed, *S* holds all SPM-relevant suffixes (represented by read numbers and read offsets) in lexicographic order of their initial *k*-mers. Let *u* be the *i*th *k*-mer in *K*. Then all SPM-relevant suffixes with common prefix *u* are stored in *S* from index *π*[*i*] to π[i+1]−1. Thus *S* can uniquely be divided into buckets of SPM-relevant suffixes with the same initial *k*-mer. Each such bucket can be sorted independently from all other buckets. Moreover, each SPM-relevant suffix not occurring in the *i*th bucket, has an initial *k*-mer different from *u* and thus cannot have a common prefix of length ≥ ℓ_*min*_ with the suffixes in the *i*th bucket. As a consequence, all suffix-prefix matches are derivable from pairs of SPM-relevant suffixes occurring in the same bucket. Thus, the suffix-prefix matches problem can be divided into *d* subproblems, each consisting of the computation of suffix-prefix matches from a bucket of SPM-relevant suffixes. This problem is considered later.

To sort the *i*th bucket one extracts the remaining suffixes relevant for sorting the bucket and stores them in a table. This strategy minimizes the number of slow random accesses to the reads. Consider the *i*th bucket and let (*p*, *q*) be one of the suffixes in the bucket, referring to the suffix of read *r*_*p*_ at read offset *q*. Then extract the suffix of *r*_*p*_ starting at position *q* + *k*. As the maximum read length is considered to be constant, the total size of the remaining suffixes to be stored is O(π[i+1]−π[i]). The remaining suffixes can be sorted using radixsort in O(π[i+1]−π[i]) time. An additional linear time scan over the sorted suffixes of the bucket delivers a table *L* of size π[i+1]−π[i]−1, such that *L*_*j*_ is the length of the longest common prefix of the suffixes S[π[i]+j−1] and *S*[*π*[*i*] + *j*] for all *j*, 1 ≤ j ≤ π[i+1]−π[i]−1.

Sorting all remaining suffixes and computing the lcp-table *L* thus requires *O*(*β*_*max*_) space and $O\left(\sum_{i=0}^{d-1}(\pi [i+1]-\pi [i])\right)=O(g)$ time where *β*_*max*_ is the maximum size of a bucket and *g* is the total number of SPM-relevant suffixes. The bucket of sorted SPM-relevant suffixes and the corresponding table *L* are processed by Algorithm 2 described after the following implementation section and Algorithm 3 described in Additional file 1, Section 7.

All in all, our algorithm runs in *O*(*m* + *n* + *g*) = *O*(*n*) time and *O*(*m* + 4^*k*′^ + 4^*k*′′^ + *β*_*max*_ + *g* + *n*) space. As we choose *k*′′ ≤ *k*′ ∈ *O*(*log*_2 _*n*) and *m*, *g*, and *β*_*max*_ are all smaller than *n*, the space requirement is *O*(*n*). Thus the algorithm for identifying and sorting all (ℓ_*min*_, *k*)-SPM-relevant suffixes is optimal.

#### Implementation

We will now describe how to efficiently implement the algorithm described above. An essential technique used in our algorithm are integer codes for *k*-mers. These are widely used in sequence processing. As we have three different mer-sizes (*k*, *k*′, and *k″*) and dependencies between the corresponding integer codes, we shortly describe the technique here. In our problem, a *k*-mer always refers to a sequence of which it is a prefix. Therefore, we introduce integer codes for strings of length ≥ *k*: For all strings *s* of length at least *k* define the integer code ϕ$\phi_{k}(s)=\sum_{i=1}^{k}\;4^{k-i}$, where *ϕ* is the mapping [*A* → 0, *C* → 1, *G* → 2, *T* → 3] uniquely assigning numbers from 0 to 3 to the bases in the alphabetical order of the bases. Note that only the first *k* symbols of *s* determine *ϕ*_*k*_(*s*), which is an integer in the range [0…4^*k*^ − 1]. For all strings *s* and *s*′ of length at least *k*, *s* ⊴ *s*′ implies *ϕ*_*k*_(*s*) ≤ *ϕ*_*k*_(*s*′), where ⊴ denotes the lexicographic order of strings and ≤ denotes the order of integers.

Besides *ϕ*_*k*_, we use the encodings *ϕ*_*k*′_ and ϕ$\phi_{k\prime \prime }^{k}$ for some *k*′, *k* ″≤ *k*. *ϕ*_*k*′_ encodes the prefix of *s* of length *k*′ and is defined in analogy to *ϕ*_*k*_ (replacing *k* by *k*′). ϕ$\phi_{k\prime \prime }^{k}(s)$ encodes the suffix s[k−k′′+1…k] of *s*[1…*k*] of length *k*″, i.e. ϕ$\phi_{k\prime \prime }^{k}(s)=\sum_{i=1}^{k\prime \prime }\;4^{k\prime \prime -i}\phi (s[k-k\prime \prime +i])$. *ϕ*_*k*′_(*s*) and ϕ${}_{k\prime \prime }^{k}(s)$ can be computed from *ϕ*_*k*_(*s*) according to the following equations:

(1) $$\phi_{k^{\prime }}(s)=\frac{\phi_{k}(s)}{4^{k-k'}}$$

(2) $$\phi_{k\prime \prime }^{k}(s)=\phi_{k}(s)\text{mod}\;4^{k^{\prime \prime }}$$

We implement *k*-mers by their integer codes. Each integer code can be computed in constant time by extracting the appropriate sequence of consecutive bit pairs from a 2bit per base encoding of the read set. In our implementation, we use the representation and the appropriate access functions from the *GtEncseq* software library [16]. As $k\leq \frac{\omega }{2}$ we can store each integer code in an integer of the machine’s word size. We sort *m* integer codes for the initial *k*-mers using quicksort, adapting the code from [17]. Our implementation works without recursion and uses an extra stack of size *O*(*log*_2 _*m*) to sort *m* integers. This small additional space requirement is the main reason to choose quicksort instead of radixsort, which is usually more than twice as fast, but requires *O*(*m*) extra working space, which we do not want to afford.

The sets *P* and *Q* are implemented by bit vectors *v*_*P*_ and *v*_*Q*_ of 4^*k*′^ and 4^*k*′′^ bits, respectively. Bit *v*_*P*_*q* is set if and only if *q* = *ϕ*_*k*′_(*r*) for some r∈R. Bit *v*_*Q*_*q* is set if and only if $q=\phi_{k^{\prime \prime }}^{k}(r)$ for some read r∈R. To obtain the bit index, one computes *ϕ*_*k*′_(*s*) and $\phi_{k\prime \prime }^{k}(s)$ from *ϕ*_*k*_(*s*) using Equations (1) and (2). Equation (1) can be implemented by a bitwise right shift of 2(*k* − *k*′) bits. Equation (2) can be implemented by a bitwise and operation with the integer 2^2*k*′′^ − 1. Thus, given the integer code for *s*, both *ϕ*_*k*′_(*s*) and *ϕ*_*k*″_^*k*^(*s*) can be computed in constant time. Therefore, the sets *P* and *Q* can be constructed in *O*(*m*) time and each access takes constant time.

When determining the *k*-mer codes in the counting phase and in the insertion phase, we sweep a window of width *k* over the sequence reads and compute the integer code for the sequence in the current window in constant time.

We implement the counts by a byte array of size *d* and store counts larger than 255 in an additional hash table. Additional file 1, Section 1 gives the details.

The partial sums in table *π* are bounded by *g*, the number of SPM-relevant suffixes. For large read sets, *g* can be larger than 2^32^ − 1. However, as the partial sum are strictly increasing, one can implement *π* by a 32 bit integer table *PS* of size *d* + 1, such that *PS*[*i*] = *π*[*i*] mod 2^32^ for any *i*, 0 ≤ *i* ≤ *d* and an additional integer table of size 2max{0,log2 g−32}marking the boundaries of carry bits. Details are given in Additional file 1, Section 2.

For the insertion phase we need a representation of the read set (2*n* bits), table *K* (2*kd* bits), set *P* and *Q* (4^*k*′^ and 4^*k*′′^ bits, respectively), table *π* (32(*d* + 1) bits) and table *S* of size *g*. As *S* holds pairs of read numbers and read offsets, each entry in *S* is stored compactly in σ=log2 m+log2(λmax−min) bits. This would give an integer array of size $\left\lceil \frac{g\;\sigma }{\omega }\right\rceil$ if we would store *S* completely. But we do not, as we employ a partitioning strategy, explained next.

Although the data structures representing tables *S*, *K*, *P* and *π* are of different sizes, their access follows the same scheme: Suppose that *i* is the smallest index, such that $\frac{g}{2}\leq \pi [i]$. Roughly half of the suffixes to be inserted in *S* are placed in buckets of lower order (with index ≤ *i*) and the other half are placed in buckets of higher order (with index > *i*). The buckets of lower order are associated with the *k*-mers in *K* up to index *i*. Hence, for these, one needs table *K* and *PS* only up to index *i*. Let *s* be some suffix of length ≤ ℓ_*min*_ such that *ϕ*_*k*_(*s*) ≤ *K*[*i*]. To apply the *P*-filter to *s*, one checks *v*_*P*_ at index $\frac{\phi_{k}(s)}{4^{k-k^{\prime }}}\leq \frac{K[i]}{4^{k-k^{\prime }}}$, which is in the first half of vector *v*_*P*_. This strategy, dividing tables *S*, *K*, *P* and *π* into *q* = 2 parts of roughly the same size, can be generalized to *q* > 2 parts. Each part is defined by a lower and an upper integer code and by corresponding lower and upper boundaries referring to sections of the four mentioned tables. Partitioning *S* means to only allocate the maximum space for holding all buckets belonging to a single part.

The four tables that can be split over *q* parts require *h*(*g*, *k*, *d*, *k*′′, *σ*) = 2*kd* + 4^*k*″^ + 32(*d* + 1) + *g σ* bits. Hence, in the insertion phase, our method requires $2n+4^{k\prime \prime }+\frac{h(g,k,d,k\prime \prime ,\sigma )}{q}$ bits, where 2*n* + 4^*k*′′^ bits are for the representation of the reads and the set *Q* (which cannot be split). As *gσ* dominates all other terms, *h*(*g*, *k*, *d*, *k*′′, *σ*) is much larger than 2*n* + 4^*k*′′^ so that the space gain of our partitioning strategy is obvious. As the space required for the insertion phase for any number of parts can be precalculated, one can choose a memory limit and calculate the minimal number of parts such that the limit is not exceeded. In particular, choosing the space peak of the counting phase as a memory limit for the insertion phase allows for balancing the space requirement of both phases. More details on the partitioning technique are given in Additional file 1, Section 3.

An obvious disadvantage of the partitioning strategy (with, say *q*, parts) is the requirement of *q* scans over the read set. However, the sequential scan over the read set is very fast in practice and only makes up for a small part of the running time of the insertion phase.

The expected size of a bucket to be sorted after the insertion phase is smaller than the average read length. The maximum bucket size (determining the space requirement for this phase) is 1 to 2 orders of magnitude smaller than *d*. As we can store $\frac{\omega }{2}$ bases in one integer of *ω* bits, the remaining suffixes (which form the sort keys) can be stored in $\beta_{\max}\left(\frac{\lambda_{\text{max}}-k}{\omega }+2\right)$ integers, where *β*_*max*_ is the maximum size of a bucket and *λ*_max_ is the maximum length of a read. The additional constant 2 is for the length of the remaining suffix, for the read number and the read offset. The sort keys are thus sequences of integers of different length which have to be compared up to the longest prefix of the strings they encode. We use quicksort in which $\frac{\omega }{2}$ bases are compared using a single integer comparison. As a side effect of the comparison of the suffixes, we obtain the longest common prefix of two compared suffixes in constant extra time, and store this in a table *L* of the size of the bucket. The suffixes in the bucket and the table *L* are passed to Algorithm 2, described next, and to Algorithm 3( Additional file 1, Section 7).

#### An efficient algorithm for computing suffix-prefix matches from buckets of sorted SPM-relevant suffixes

The input to the algorithm described next is a bucket of sorted SPM-relevant suffixes, with the corresponding table *L*, as computed by the algorithm of the previous subsection. Consider the *i*th bucket in *S* and let *H*_*j*_ = *S*[*π*[*i*] *+ j*] be the *j*th suffix in this bucket for all *j*, 0 ≤ *j* ≤ *β* − 1 where *β* = *π*[*i* + 1] − *π*[*i*] is the size of the bucket. By construction, we have *H*_*j*-1_ ⊴ *Hj*, *L*_*j*_ ≥ *k*, and *L*_*j*_ is the length of the longest common prefix of *H*_*j*−1_ and *H*_*j*_ for *j*, 1 ≤ *j* ≤ *β* − 1.

Note that the bucket-wise computation does not deliver the lcp-values of pairs of SPM-relevant suffixes on the boundary of the buckets. That is, for all *i* > 0, the length of the longest common prefix of *S*[*π*[*i*] − 1] and *S*[*π*[*i*]] is not computed, because *S*[*π*[*i*] − 1] is the last suffix of the (*i* − 1)th bucket and *S*[*π*[*i*]] is the first suffix of the *i*th bucket. However, as both suffixes belong to two different buckets, their longest common prefix is smaller than *k* (and thus smaller than ℓ_*min*_) and therefore not of interest for the suffix-prefix matching problem.

The suffixes occurring in a bucket will be processed in nested intervals, called lcp-intervals, a notion introduced for enhanced suffix arrays by [18]. We generalize this notion to table *H* and *L* as follows: An interval *e*.*f*, 0 ≤ *e* < *f* ≤ *β* − 1, is an *lcp-interval of lcp-value* ℓ if the following holds:

 • 

(3) $$e=0\;\text{or}L_{e}<|\text{,}$$

 • 

(4) Lq≥|for all q,e+1≤q ≤f,

 • 

(5) $$L_{q}=|\;\text{for at least one}q,e+1\leq q\leq f\text{,}$$

 • 

(6) $$f=\beta -1\;\text{or}L_{f+1}<|\text{.}$$

We will also use the notation ℓ − [*e*.*f*] for an lcp-interval [*e*.*f*] of lcp-value ℓ. If ℓ − [*e*.*f*] is an lcp-interval such that *w* = *H*_*e*_[1…ℓ] is the longest common prefix of the suffixes *H*_*e*_, *H*_*e*+1_, …, *H*_*f*_, then [*e*.*f*] is called the *w*-interval.

An lcp-interval ℓ′ − [*e*′.*f*′] is said to be *embedded* in an lcp-interval ℓ − [*e*.*f*] if it is a proper subinterval of ℓ − [*e*.*f*] (i.e., *e* ≤ *e*′ < *f*′ ≤ *f*) and ℓ′ > ℓ. The lcp-interval ℓ − [*e*.*f*] is then said to be *enclosing* [*e*′.*f*′]. If [*e*.*f*] encloses [*e*′.*f*′] and there is no interval embedded in [*e*.*f*] that also encloses [*e*′.*f*′], then [*e*′.*f*′] is called a *child interval* of [*e*.*f*] and [*e*.*f*] is the *parent interval* of [*e*′.*f*′]. We distinguish lcp-intervals from singleton intervals [*e*′] for any *e*′, 0 ≤ *e*′, ≤ *β* − 1. [*e*′] represents *H*_*e*′_. The parent interval of [*e*′] is the smallest lcp-interval [*e*.*f*] with *e* ≤ *e*′ ≤ *f*.

This parent–child relationship of lcp-intervals with other lcp-intervals and singleton intervals constitutes a virtual tree which we call the *lcp-interval tree* for *H* and *L*. The root of this tree is the lcp-interval 0 − [0.(*β* − 1)]. The implicit edges to lcp-intervals are called *branch*-edges. The implicit edges to singleton-intervals are called *leaf*-edges. Additional file 1, Section 10 gives a comprehensive example illustrating these notions.

**Figure 1:**
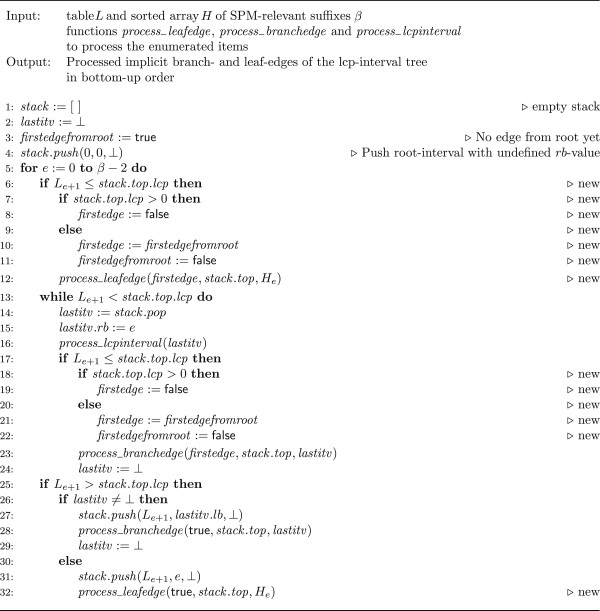
**Algorithm 1.** Bottom-up traversal algorithm for arrays of SPM-relevant suffixes. This is an extension of [18, Algorithm 4.4] with the additional lines marked as new.

Abouelhoda et al. ([18], Algorithm 4.4) present a linear time algorithm to compute the implicit branch-edges of the lcp-interval tree in bottom-up order. When applied to a bucket of sorted suffixes, the algorithm performs a linear scan of tables *H* and *L*. In the *e*th iteration, 0 ≤ *e* ≤ *β* − 2, it accesses the value *L*_*e*+1_ and *H*_*e*_. We have non-trivially extended the algorithm to additionally deliver leaf edges. The pseudocode, with some additions in the lines marked as *new*, is given in Algorithm 1 (Figure 1). We use the following notation and operations:

 • A stack stores triples (ℓ, *e*, *f*) representing an lcp-interval ℓ − [*e*.*f*]. To access the elements of such a triple, say *sv*, we use the notation *sv*.*lcp* (for the lcp-value ℓ), *sv*.*lb* (for the left boundary *e*) and *sv*.*rb* (for the right boundary *f*).

 • *stack*.*push*(*e*) pushes an element *e* onto the stack.

 • *stack*.*pop* pops an element from the stack and returns it.

 • *stack*.*top* returns a reference to the topmost element of the stack.

 • ⊥ stands for an undefined value.

 • *process*_*leafedge*(*firstedge*, *itv*, (*p*, *q*)) processes an edge from the lcp-interval *itv* to the singleton interval representing the suffix *r*_*p*_[*q*…|*r*_*p*_|]. *firstedge* is true if and only if the edge is the first processed edge outgoing from *itv*.

 • *process*_*branchedge*(*firstedge*, *itv*, *itv’*) processes an edge from the lcp-interval *itv* to the lcp-interval *itv’*. The value *itv’*.*rb* is defined and *firstedge* is true if and only if the edge is the first edge outgoing from *itv*.

 • *process*_*lcpinterval*(*itv*) processes the lcp-interval *itv*. The value *itv*.*rb* is defined.

Depending on the application, we use different functions *process*_*leafedge*, *process*_*branchedge*, and *process*_*lcpinterval*.

Additional file 1, Section 4, explains why Algorithm 1 also delivers the leaf edges of the lcp-interval tree in the correct bottom-up order.

Consider a path in the lcp-interval tree from the root to a singleton interval [*e*′] representing *H*_*e*′_ = *r*_*p*_[*q*…|*r*_*p*_|]. Let ℓ − [*e*.*f*] be an lcp-interval on this path, and consider the edge on this path outgoing from ℓ − [*e*.*f*]. If the edge goes to an lcp-interval of, say lcp-value ℓ′, then the edge is implicitly labeled by the non-empty sequence $r_{p}[q+l\ldots q+l\prime -1]$. Suppose the edge goes to a singleton interval: Then the edge is implicitly labeled by the non-empty sequence *r*_*p*_[*q* + ℓ…|*r*_*p*_| − 1]$_*p*_. If *q* + ℓ = |*r*_*p*_|, then *r*_*p*_[*q* + ℓ…|*r*_*p*_| − 1] is the empty string, which implies that the edge to the singleton interval is labeled by the sentinel $_*p*_. Such an edge is a *terminal edge* for *r*_*p*_. If the read offset *q* is 0, we call [*e*′] a *whole*-*read interval* for *r*_*p*_, and the path in the lcp-interval tree from the root to [*e*′] a *whole*-*read path* for *r*_*p*_.

**Figure 2:**
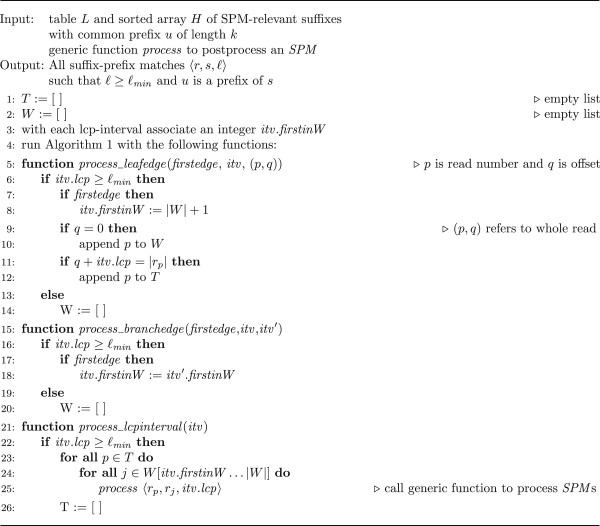
**Algorithm 2.** Bottom-up traversal of lcp-interval tree enumerating suffix-prefix matches.

Consider a suffix-prefix match 〈 *r*_*p*_, *r*_*j*_, ℓ 〉 , such that the suffix *w* of *r*_*p*_ of length ℓ has a prefix *u* of length *k*. Recall that *u* is the common prefix of all suffixes in the *i*th bucket. Due to the implicit padding of reads at their end, the symbol following *w* as a suffix of *r*_*p*_ is $_*p*_. By definition, *w* is also prefix of *r*_*j*_ and the symbol in *r*_*j*_ following this occurrence of *w* is different from $_*p*_. Thus, there is a *w*-interval [*e*.*f*] in the lcp-interval tree for *H* and *L*. [*e*.*f*] is on the path from the root-interval to the whole-read leaf interval for *r*_*j*_. Moreover, there is a terminal edge for *r*_*p*_ outgoing from [*e*.*f*]. Vice versa, an lcp-interval of lcp-value ℓ on the path to the whole-read leaf interval for *r*_*j*_ and with a terminal edge for *r*_*p*_ identifies the suffix-prefix match 〈 *r*_*p*_, *r*_*j*_, ℓ 〉 . This observation about suffix-prefix matches is exploited in Algorithm 2 (Figure 2) which performs a bottom-up traversal of the lcp-interval tree for *H* and *L*, collecting whole-read leaves and terminal edges for lcp-intervals of lcp-value at least ℓ_*min*_. More precisely, whenever a whole-read leaf for *r*_*p*_, 1 ≤ *p* ≤ *m*, is found (line 9), *p* is appended to the list *W*. With each lcp-interval *itv* on the stack used in the bottom-up traversal, an integer *itv*.*firstinW* is associated. The elements in *W*[*itv*.*firstinW*…|*W*|] are exactly the read numbers of whole-read leaves collected for *itv*. The value of *itv*.*firstinW* is set whenever the first edge outgoing from *itv* is detected: If the first edge outgoing from *itv* is a leaf-edge, no previous whole-read leaf for *itv* has been processed: Thus |*W*| + 1 is the first index in list *W* where the whole read leaf information (if any) for *itv* will be stored (see line 8). If the first edge is a branch-edge to lcp-interval *itv*′, then the corresponding subset of *W* for *itv*′ must be inherited to *itv*. Technically, this is achieved by inheriting the *firstinW*-attribute from *itv*′ to *itv*, see line 18 of Algorithm 2.

Whenever a terminal edge for read *r*_*p*_, outgoing from an interval *itv* is processed (line 11), *p* is added to the list *T*. Suppose that this terminal edge is outgoing from the lcp-interval *itv*. The first symbol of the label of the terminal edge is $_*p*_. Suppose there is a branch-edge outgoing from *itv* to some lcp-interval *itv*′. Then the first symbol, say *a*, of the implicit label of this edge must occur more than once. Thus it cannot be a sentinel, as these are considered different in the lexicographic ordering of the suffixes. Hence the first symbol *a* is either *A*, *C*, *G* or *T*. As these symbols are, with respect to the lexicographic order, smaller than the sentinels, the branch-edge from *itv* to *itv*’ appears before the terminal edge from *itv*. Hence the terminal edges outgoing from *itv*′ have been processed in line 25, and so we only need a single list *T* for the entire algorithm.

As soon as all edges outgoing from *itv* have been processed, we have collected the terminal edges in *T* and the whole-read leaves in *W*. If *itv*.*lcp* exceeds the minimum length, Algorithm 2 computes the cartesian product of *T* with the appropriate subset of *W* and processes the corresponding suffix-prefix matches of length *itv*.*lcp* in line 25. At this point suffix-prefix matches may be output or post-processed to check for additional constraints, such as transitivity. Once the cartesian product has been computed, the elements from *T* are no longer needed and *T* is emptied (line 26). Note that the algorithm empties *W* once an lcp-interval of lcp-value smaller than ℓ_*min*_ is processed. After this event, there will only be terminal edges from *v*-intervals such that the longest common prefix of *v* and the reads in *W* is smaller than ℓ_*min*_. Therefore there will be no suffix-prefix match of the form 〈_, *w*, ℓ〉 such that ℓ ≥ ℓ_*min*_ and *w* is a read represented in *W*. So the list can safely be emptied.

The lcp-interval tree for *H* and *L* contains *β* leaf-edges. As all lcp-intervals have at least two children, there are at most *β* − 1 branch-edges and *β* lcp-intervals. As each of the three functions specified in Algorithm 2 is called once for every corresponding item, the number of functions calls is at most 3*β* − 1. Recall that Algorithm 2 is applied to each bucket and the total size of all buckets is *g*. Hence there are at most 3*g* − *d* calls to the three functions. *process*_*leafedge* and *process*_*branchedge* run in constant time. The running time of *process*_*lcpinterval* is determined by the number of *SPM*s processed. Assuming that the processing takes constant time, the overall running time of Algorithm 2 for all buckets is *O*(*g* + *z*) where *z* is the number of processed *SPM*s.

#### Handling reverse complements of reads

Reads may originate from both strands of a DNA molecule. For this reason, suffix-prefix matches shall also be computed between reads and reverse complements of other reads. Handling the reverse complements of all reads is conceptually easy to integrate into our approach: One just has to process $\overset{―}{R}$ instead of R.

The three steps which involve scanning the reads are extended to process both strands of all reads. This does not require doubling the size of the read set representation, as all information for the reverse complemented reads can efficiently be extracted from the forward reads. Additional file 1, Section 5, shows how to compute the integer codes for the reversed reads from the integer codes of the forward reads in constant time.

The scan of the reverse complemented reads has a negligible impact on the runtime. Of course, the size of the table *S*, *K* and *PS* roughly doubles when additionally considering reverse complements.

When computing suffix-prefix matches some minor modifications are necessary: Applying Algorithm 2 to $\overset{―}{R}$ finds all *SPM*s, including some redundant ones, which we want to omit. This is formalized as follows: an *SPM*$〈r,s,l〉\in SPM(\overset{―}{R})$ is *non-redundant* if and only if one of the following conditions is true:

 • 

(7) r∈R,s∈R

 • $r\in R,s\in \overset{―}{R},r≺\overset{―}{s}$

 • $r\in \overset{―}{R},s\in R,s≺\overset{―}{r}\text{.}$

For any *SPM*, these conditions can easily be checked in constant time, see Algorithm 3 ( Additional file 1, Section 7).

### Recognition of transitive and irreducible suffix-prefix matches

**Figure 3:**
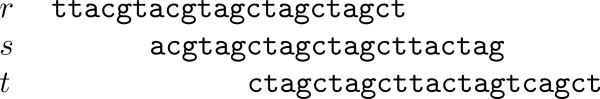
**Example of a transitive suffix-prefix match.** An example of a transitive SPM. A set of three reads with a transitive SPM 〈*r*, *t*, 10〉 derived from 〈*s*, *t*, 16〉.

For the construction of the string graph, we do not need transitive *SPM*s. An *SPM*$〈r,t,l\prime \prime 〉$ is *transitive* if and only if there are two *SPM*s 〈*r*, *s*, ℓ〉 and 〈*s*, *t*, ℓ′〉 such that ℓ + ℓ′ = |*s*|+ ℓ″. Figure 3 shows a concrete example of a transitive *SPM*. An *SPM* which is not transitive is *irreducible*.

**Figure 4:**
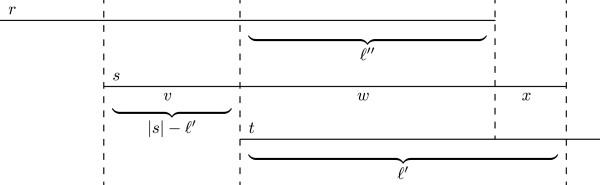
**Illustration of transitivity of a suffix-prefix match.** Schematic illustration of transitivity. $〈r,t,l\prime \prime 〉$ is a transitive SPM derived from the SPM 〈*s*, *t*, ℓ’〉. Hence, the prefix v of s is a suffix of r[1…|r|−l′′].

The following theorem characterizes an *SPM* by a read and a single irreducible *SPM* satisfying a length constraint and a match constraint, see Figure 4 for an illustration.

**Theorem 1.** Let 〈*r*, *t*, ℓ″〉 be an *SPM*. Then 〈*r*, *t*, ℓ″〉 is transitive if and only if there is an s∈R and an irreducible *SPM* 〈*s*, *t*, ℓ′〉 such that ℓ′ > ℓ″, |*r*| − ℓ″ ≥ |*s*| − ℓ′ and *s*[1…|*s*| − ℓ′] = *r*[|*r*| − ℓ″ − (|*s*| − ℓ′) + 1…|*r*| − ℓ″].

The proof of Theorem 1 can be found in Additional file 1, Section 6.

If the *SPM* 〈*r*, *t*, ℓ″〉 is transitive and 〈*s*, *t*, ℓ′〉 is the *SPM* satisfying the conditions of Theorem 1, then we say that 〈*r*, *t*, ℓ″〉 *is derived from* 〈*s*, *t*, ℓ′〉.

Theorem 1 suggests a way to decide the transitivity of an *SPM* 〈*r*, *t*, ℓ〉: Check if there is an irreducible *SPM* 〈*s*, *t*, ℓ′〉 from which it is derived. The check involves comparison of up to |*s*| − ℓ′ symbols to verify if *s*[1…|*s*| − ℓ′] is a suffix of r[1…|r|−l′′]. As there may be several irreducible *SPM*s from which 〈*r*, *t*, ℓ″〉 may be derived, it is necessary to store the corresponding left contexts: For any sequence *s* and any ℓ′, 1 ≤ ℓ′ < |*s*|, the *left context LC*(*s*, ℓ′) of *s* is the non-empty string *s*[1…|*s*| − ℓ′].

Due to the bottom-up nature of the traversal in Algorithm 2, the *SPM*s involving the different prefixes of a given read are enumerated in order of match length, from the longest to the shortest one. Thus, Algorithm 2 first delivers the irreducible *SPM* 〈*s*, *t*, ℓ′〉 from which $〈r,t,l\prime \prime 〉$ is possibly derived, because l′>l′′.

**Figure 5:**
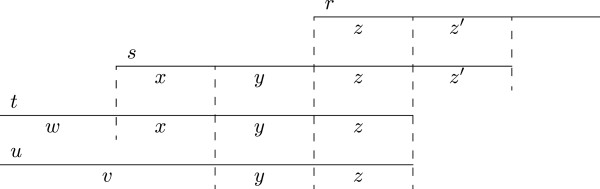
**Transitivity and left contexts.** Transitivity and left contexts. Let the SPM 〈*t*, *r*, |*z*|〉 be derived from 〈*s*, *r*, |*zz*’|〉. Hence the left context *LC*(*s*, |*zz*’|) = *xy* is a suffix of the left context *LC*(*t*, |*z*|) = *wxy*. Let 〈*u*, *r*, |*z*|〉 be an irreducible SPM. Then *LC*(*u*, |*yz*|) = *vy* for some non empty string *v* and *LC*(*s*, |*zz*’|) is not a suffix of *vy*.

From Theorem 1 one can conclude that the first *SPM*, say 〈*s*, *t*, ℓ′〉, found on a whole-read path for *t* is always irreducible. Hence, one stores *LC*(*s*, ℓ′). An *SPM* 〈*r*, *t*, ℓ″〉 detected later while traversing the same whole-read path for *t* is classified as transitive if and only if *LC*(*s*, ℓ′) is a suffix of *LC*(*r*, ℓ″) (see Figure 5 for an illustration). If 〈*r*, *t*, ℓ″〉 is transitive it is discarded. Otherwise, *LC*(*r*, ℓ″) must be stored as well to check the transitivity of the *SPM*s found later for the same whole-read path. So each *SPM* is either classified as transitive, or irreducible, in which case a left context is stored. To implement this method, we use a dictionary *D* of left contexts, with an operation *LCsearch*(*D*, *s*), which returns *true* if there is some *t* ∈ *D* such that *t* is a suffix of *s*. Otherwise, it adds *s* to *D* and returns *false*. Such a dictionary can, for example, be implemented by a trie [19] storing the left contexts in reverse order. In our implementation we use a blind-trie [20]. In Additional file 1, Section 7 we present a modification of Algorithm 2 to output non-redundant irreducible *SPM*s only.

### Recognition of internally contained reads

At the beginning of the methods section we have shown how to detect reads which are prefixes or suffixes of other reads. When constructing the string graph we also have to discard internally contained reads, which are contained in other reads without being a suffix or a prefix. More precisely, r∈R is *internally contained*, if a read r′∈R exists, such that *r*′ = *urw* for some non-empty strings *u* and *v*. In Additional file 1, Section 8, we show how to efficiently detect internally contained reads.

### Construction of the assembly string graph

Consider a read set R which is suffix- and prefix-free. The assembly string graph [8] is a graph of the relationships between the reads, constructed from $SPM^{\mathrm{nr}}(\overset{―}{R})$, the set of all non-redundant irreducible *SPM*s from $SPM(\overset{―}{R})$ restricted to reads which are not internally contained.

For each r∈R the graph contains two vertices denoted by *r*.*B* and *r*.*E*, representing the two extremities of the read. *B* stands for begin, *E* stands for end.

For each non-redundant irreducible *SPM*$〈r,s,l〉\in SPM^{\mathrm{nr}}(\overset{―}{R})$ satisfying ℓ ≥ ℓ_*min*_, the graph contains two directed edges, defined as follows:

1. if $〈r,s,l〉\in SPM^{\mathrm{nr}}(\overset{―}{R})$ there are two edges:

 • *r*.*E* → *s*.*E* with edge label *s*[ℓ + 1…|*s*|]

 • *s*.*B* → *r*.*B* with edge label $\bar{r}[l+1\ldots |r|]$

2. if $〈r,\bar{s},l〉\in SPM^{\mathrm{nr}}(\bar{R})$ there are two edges:

 • *r*.*E* → *s*.*B* with edge label $\bar{s}[l+1\ldots |s|]$

 • *s*.*E* → *r*.*B* with edge label $\bar{r}[l+1\ldots |r|]$

3. if $〈\overset{―}{s},r,l〉\in SPM^{\mathrm{nr}}(\bar{R})$ there are two edges:

 • *r*.*B* → *s*.*E* with edge label *s*[ℓ + 1…|*s*|]

 • *s*.*B* → *r*.*E* with edge label *r*[ℓ + 1…|*r*|]

In our implementation of the string graph, vertices are represented by integers from 0 to 2*m* − 1. To construct the graph from the list of non-redundant irreducible *SPM*s, we first calculate the outdegree of each vertex. From the counts we calculate partial sums. In a second scan over the list of *SPM*s, we insert the edges in an array of size 2*ρ*, where $\rho =|SPM^{\mathrm{nr}}(\bar{R})|$. This strategy allows to allocate exactly the necessary space for the edges and to access the first edge outgoing from a vertex in constant time. The array of edges is stored compactly using $2\rho (\left\lceil log_{2}(2m)\right\rceil +\left\lceil log_{2}(\lambda_{\text{max}}-l_{\min})\right\rceil )$ bits, where *λ*_max_ is the maximum length of a read. $\left\lceil log_{2}(2m)\right\rceil$ bits are used for the destination of an edge (the source of the edge is clear from the array index where the edge is stored). $\left\lceil log_{2}(\lambda_{\text{max}}-l_{\min})\right\rceil$ bits are used for the length of the edge label.

To output the contigs, we first write references (such as read numbers and edge lengths) to a temporary file. Once this is completed, the memory for the string graph is deallocated, and the read sequences are mapped into memory. Finally, the sequences of the contigs are derived from the references and the contigs are output.

To verify the correctness of our string graph implementation and to allow comparison with other tools, we have implemented the graph cleaning algorithms described in [9] as an experimental feature. More sophisticated techniques, such as the network flow approach described in [8], are left for future work, as the main focus of this paper lies in the efficient computation of the irreducible *SPM*s and the construction of the string graph.

## Results

The presented methods for constructing the string graph and the subsequent computation of contigs have been implemented in a sequence assembler named *Readjoiner*, which is part of the *GenomeTools* software suite [21]. The *Readjoiner* pipeline consists of three steps:

 • *Readjoiner prefilter* takes a read set in form of one or more Fasta-formatted files and removes reads containing ambiguity codes and reads which are prefixes or suffixes of other reads. It outputs the prefiltered reads in the *GtEncseq*-format [16].

 • *Readjoiner overlap* maps the representation of prefiltered reads in memory, enumerates non-redundant irreducible suffix-prefix matches, and stores them on file. The time/space tradeoff for this step can be adjusted by an option specifying the number of parts in which the sorted array of SPM-relevant suffixes is computed. Alternatively, one can specify a memory limit, according to which the minimum number of parts is determined to not exceed this limit.

 • *Readjoiner assembly* builds the string graph and traverses it to output the contigs.

### Experimental setup

For our benchmarks, the 64bit *GenomeTools* binary was compiled by *gcc* version 4.3.2 using the provided Makefile with the option “64bit = yes assert = no amalgamation = yes”. These last two options trigger the build-system to not compile assertions and to generate a single C-code file from which an amalgamation object is compiled, thus allowing for a maximum of inlined code, which in turn is executed faster.

All tests were performed on a computer with a 2.40 Ghz Intel Xeon E5620 4-core processor, 64 GB RAM, under a 64bit Linux operating system, using a single core only.

For memory usage measurements, we monitored the VmHWM (“high water mark”) value in the /proc file system [22] associated with the process of each particular program over the time of its execution, including both allocated heap memory and memory made available via the mmap() system call. The running time is the CPU time (sum of user and system time) as measured using *GNU time*.

For all runs of *Readjoiner* we used *k* = 32, $k\prime =max\{8,\left\lceil log_{2}n\right\rceil -8\}$ and *k″* = *k*′ − 1. If not stated otherwise, the number of parts in which *Readjoiner* computes the sorted array of SPM-relevant suffixes was 7. All programs were run with _*min*_ = 45 (which is the default minimum match length in SGA).

### Human genome sequencing simulations

We tested our assembler on simulated error-free sequencing read sets based on human genomic sequences (latest available release of GRCh37). For each human chromosome we prepared a template sequence by deleting ambiguity symbols. Then we simulated reads by pseudo random sampling of the template sequence and its reverse complement, until the desired number of reads was obtained. This was done using the *GenomeTools simreads*-tool and is the same procedure as used in [11,13].

From each of the 24 human chromosome sequences, we generated a separate read set with 20 × coverage and a constant read length of 100 bp. The read set are called *c*1, *c*2, …, *c*22, *cX*, *cY*. Furthermore, using the entire human genome as template we generated read sets with 20×, 30× and 40× coverage, referred to by hg20×, hg30× and hg40×, respectively. Additionally, from chromosome 22, a set of reads of variable length was prepared: The results for this dataset are reported in Additional file 1, Section 9.

**Figure 6:**
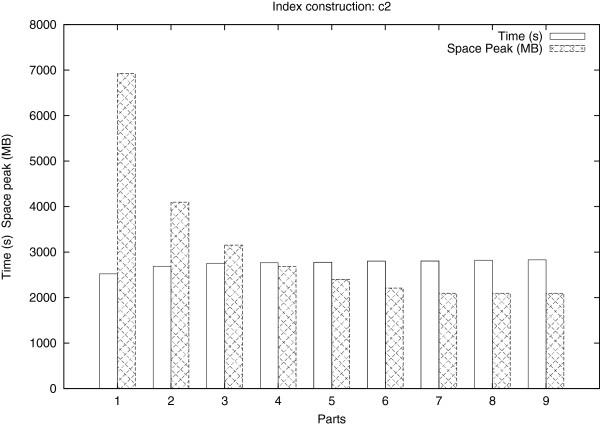
**Influence of the partitioning technique on space and time requirement.** Influence of the partitioning technique on space and time requirement. Running time and space peak of Readjoiner for the index construction of the c2 dataset with a varying number of parts (from 1 to 9, ℓ_*min*_ = 45).

In a first computational experiment, we determined the time vs. space tradeoff of our partitioning strategy, by applying *Readjoiner* to c2 with a varying number of parts ranging from 1 to 9. The results are shown in Figure 6.

**Figure 7:**
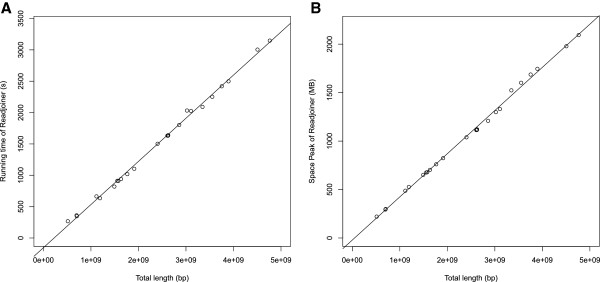
**Running time and space peak for Readjoiner for all 24 read sets derived from human chromosomes.** Running time (**A**) and space peak (**B**) of Readjoiner for all 24 read sets c1, c2, …, c22, cX, cY derived from the human chromosomes (ℓ_*min*_ = 45). Each dot represents a human chromosome placed on the X-axes according to its length and on the Y-axes according to the running time (**A**) and the space peak (**B**) required by Readjoiner to process it. The line was fitted to the dots using the least square regression command lm from the R-project http://www.r-project.org/. This delivered R^2^ = 0.997 for the running time and R^2^ = 0.998 for the space peak.

The complete *Readjoiner*-pipeline was applied to each of the 24 datasets *c*1, *c*2, *c*3, …, *c*22, *cX*, *cY*. We considered the running time as a function of the length of each chromosome from which the dataset was generated and performed a linear regression, which delivered an *R*^2^-value of 0.997. The same was done for the space peak, delivering an *R*^2^-value of 0.998. Figure 7 shows plots of the time vs. length and space peak vs. length functions.

### Comparison with other string graph-based assemblers

The 64bit Linux binaries of Edena [9] were downloaded from [23]. We tested version 2.1.1 and version 3 dev110920. Edena 3 is an untested version and under active development. In our tests, it required slightly more memory and was significantly slower than Edena version 2.1.1, which we therefore selected for the comparative test. The source code of SGA (version 0.9.13) was obtained from its public GitHub repository [24]. The 64-bit Linux binary of LEAP was downloaded from [25].

As Edena is based on the original string graph construction method proposed by [8], a comparison to *Readjoiner* allows validating our construction method. Table 1 reports the performance of Edena and *Readjoiner* for the assembly of datasets c22, c15 and c7. These and additionally c2 were used as benchmark sets in [11,13]. For all three datasets, Edena and *Readjoiner* produce exactly the same list of irreducible *SPM*s, which allows concluding that the string graphs are identical. The resulting contig sets are almost identical: Edena was slightly more stringent in the output of the smallest contigs. *Readjoiner* was 13 − 14× faster than Edena and used about 11% of the space used by Edena. Due to a segmentation fault, Edena did not complete the overlap phase for the largest chromosome dataset c2.

**Table 1:** Comparison of Readjoiner and Edena

	RJ	Edena	$\frac{\text{Edena}}{\text{RJ}}$	RJ	Edena	$\frac{\text{Edena}}{\text{RJ}}$	RJ	Edena	$\frac{\text{Edena}}{\text{RJ}}$
Read set	c22	c22	-	c15	c15	-	c7	c7	-
Genome size (Mbp)	34.9	34.9	-	81.7	81.7	-	155.4	155.4	-
Number of reads (M)	7.0	7.0	-	16.3	16.3	-	31.1	31.1	-
Contained reads (K)	686.4	686.4	-	1665.7	1665.7	-	3103.0	3103.0	-
Irreducible SPM (M)	7.2	7.2	-	17.2	17.2	-	36.4	36.4	-
Overall time (s)	360	4903	13.62×	945	13609	14.40×	2035	29404	14.45×
Overall space (MB)	294	2753	9.35×	703	6415	9.13×	1331	12255	9.21×
Contigs	120712	120462	-	254830	254111	-	503446	502706	-
Total contigs length (Mbp)	45.7	44.7	-	103.0	101.1	-	198.8	195.0	-
Assembly N50 (Kbp)	1.6	1.7	-	2.4	2.5	-	2.3	2.4	-
Assembly NG50 (Kbp)	2.7	2.7	-	3.7	3.7	-	3.9	3.9	-
Longest contig (Kbp)	41.4	41.4	-	54.2	54.2	-	44.9	44.9	-

Results of applying Readjoiner (RJ) and Edena to the datasets c22, c15, c7 (ℓ_*min*_ = 45).

SGA [12] is based on the direct string graph construction methods first introduced in [11]. Currently, to the best of our knowledge, it is the only other open source string graph-based assembler, besides *Readjoiner*. The SGA default pipeline consists of the index, overlap and assemble tools: However, memory can be reduced by using sga index, rmdup and fm-merge [12]. The parameter *d* of the SGA-index phase allows selecting the number of sequences to be processed at a time: without this parameter, the index phase requires much more memory than other phases. With memory being the most limiting factor in the assembly, we optimized the space peak of SGA by gradually reducing the value of *d* until either the space peak of the index phase was less than the space peak of the other phases, or a further decrease of *d* did not reduce the space peak. SGA’s index construction and overlap calculation can be threaded. However, for a fair comparison we used a single thread. Table 2 reports the results of running SGA and *Readjoiner* for the datasets c22, c15, c7 and c2. *Readjoiner* was 19× to 20× faster than SGA and used 14% to 23% less space than SGA. Using overlap and assemble instead of fm-merge, SGA became slightly faster but required about 7 times more memory (data not shown). By default, SGA is less stringent than *Readjoiner* when computing the contigs from the string graph, thus producing more contigs with a lower N50 value. For each of the four datasets, the longest contig produced by SGA and *Readjoiner* is identical, and the NG50 value of the assembly is comparable.

**Table 2:** Comparison of Readjoiner and SGA

	RJ	SGA	$\frac{\text{SGA}}{\text{RJ}}$	RJ	SGA	$\frac{\text{SGA}}{\text{RJ}}$	RJ	SGA	$\frac{\text{SGA}}{\text{RJ}}$	RJ	SGA	$\frac{\text{SGA}}{\text{RJ}}$
Read set	c22	c22	-	c15	c15	-	c7	c7	-	c2	c2	-
Genome size (Mbp)	34.9	34.9	-	81.7	81.7	-	155.4	155.4	-	238.2	238.2	-
Number of reads (M)	7.0	7.0	-	16.3	16.3	-	31.1	31.1	-	47.6	47.6	-
Sga index -d (K)	-	300	-	-	700	-	-	1350	-	-	2300	-
Overall time (s)	360	7508	20.86×	945	19334	20.46×	2035	39988	19.65×	3185	65194	20.47×
Overall space (MB)	294	383	1.30×	703	842	1.20×	1331	1568	1.18×	2094	2436	1.16×
Contigs	120712	231594	-	254830	547217	-	503446	1215816	-	634403	1702714	-
Total contigs length (Mbp)	45.7	55.9	-	103.0	130.5	-	198.8	266.4	-	292.2	396.1	-
Assembly N50 (Kbp)	1.6	0.8	-	2.4	1.0	-	2.3	0.5	-	3.2	1.2	-
Assembly NG50 (Kbp)	2.7	2.7	-	3.7	3.7	-	3.9	3.9	-	4.5	4.5	-
Longest contig (Kbp)	41.4	41.4	-	54.2	54.2	-	44.9	44.9	-	52.9	52.9	-

Results of applying Readjoiner (RJ) and SGA to the datasets c22, c15, c7, c2 (ℓ_*min*_ = 45).

LEAP implements the methods described in [13] to construct a full overlap graph. The efficiency of LEAP is remarkable, and allowed us to extend the comparison with *Readjoiner* to the hg20× dataset. Table 3 reports the results when applying *Readjoiner* and LEAP to the datasets c22, c2 and hg20×. Additionally, it shows the results for *Readjoiner* on hg30× and hg40× (for which LEAP was not able to complete the overlap phase on our test machine with 64 GB RAM). *Readjoiner* was faster than LEAP for all datasets with a speedup factor of 1.6 to 1.8. Furthermore, it required less memory: While the reduction in the space peak was at a maximum for the small datasets (c22, 2.99×), it is still significant (1.63 × ) for the hg20× dataset which contains almost two orders of magnitude more reads. In approximately the same time in which LEAP assembles hg20× , and using less memory, *Readjoiner* was able to assemble the hg30× dataset. *Readjoiner* was also able to assemble the hg40× dataset in 51 hours using 52 GB RAM.

**Table 3:** Comparison of Readjoiner and LEAP

	RJ	LEAP	$\frac{\text{LEAP}}{\text{RJ}}$	RJ	LEAP	$\frac{\text{LEAP}}{\text{RJ}}$	RJ	LEAP	$\frac{\text{LEAP}}{\text{RJ}}$	RJ	RJ
Read set	c22	c22	-	c2	c2	-	hg20×	hg20×	-	hg30×	hg40×
Genome size (Mbp)	34.9	34.9	-	238.2	238.2	-	2861.3	2861.3	-	2861.3	2861.3
Number of reads (M)	7.0	7.0	-	47.6	47.6	-	579.5	579.5	-	869.2	1155.3
Overall time	6 min	9 min	1.60×	53 min	1 h 36 min	1.81×	20 h 4 min	35 h 56 min	1.79×	34 h 9 min	51 h 16 min
Overall space (GB)	0.3	0.9	2.99×	2.0	4.0	1.98×	27.9	45.6	1.63×	39.8	52.0
Contigs	120712	113428	-	634403	630408	-	3239309	11662607	-	13497497	16253905
Total contigs length (Mbp)	45.7	43.1	-	292.2	280.6	-	2833.1	3642.7	-	4003.9	4281.1
Assembly N50 (Kbp)	1.6	1.6	-	3.2	3.0	-	3.0	1.4	-	1.2	0.9
Assembly NG50 (Kbp)	2.7	2.4	-	4.5	3.9	-	3.0	2.5	-	2.9	2.8
Longest contig (Kbp)	41.4	39.4	-	52.9	48.9	-	63.4	58.6	-	63.4	63.4

Results of applying Readjoiner (RJ) and LEAP to the datasets c22, c2, hg20× , hg30× , hg40× (ℓ_*min*_ = 45). LEAP was not able to process hg30× and hg40× on the test machine with 64 GB RAM.

### Evaluation of assemblies

In order to assess the quality of the assemblies delivered by the different programs, we used the script assess_assembly.pl of the Plantagora project [26]. The script aligns the contigs to the template sequence from which the reads were sampled, using the Nucmer alignment tool [27]. Several metrics, including the number of unaligned contigs, misassemblies, SNPs and gaps are reported.

Furthermore, assemblies were evaluated using the basic Assemblathon 1 statistics as defined in [28], including total length of the contigs, length of the longest and shortest contig, N50, L50, NG50 and LG50. Table 4 reports the results of the evaluation of the assemblies of dataset c22.

**Table 4:** Metrics of assemblies of the c22 dataset

Assemblathon metrics	RJ	SGA	Edena	LEAP
Number of contigs	120712	231594	120462	113428
Genome size (bp)	34894545	34894545	34894545	34894545
Total contigs length	45667531	55880641	44737441	43099113
- as % of genome	130.87	160.14	128.21	123.51
Mean contig size	378.32	241.29	371.38	379.97
Median contig size	132	101	120	117
Longest contig	41352	41352	41352	39379
Shortest contig	102	100	100	101
Contigs > 500 bp	13467 (11.16%)	13416 (5.79%)	13439 (11.16%)	13430 (11.84%)
Contigs > 1 Kbp	8700 (7.21%)	8684 (3.75%)	8696 (7.22%)	8578 (7.56%)
Contigs > 10 Kbp	264 (0.22%)	264 (0.11%)	264 (0.22%)	228 (0.20%)
N50	1614	815	1699	1617
L50	5684	10118	5416	5488
NG50	2737	2739	2733	2461
LG50	3120	3113	3121	3429

**Plantagora metrics**	**RJ**	**SGA**	**Edena**	**LEAP**
Covered Bases	34343945	34357693	34300114	12968118
Ambiguous Bases	159997	154584	182952	696334
Misassemblies	4	4	4	3693
Misassembled Contigs	4	4	4	2344
Misassembled Contig Bases	1283	417	1245	2797710
SNPs	104	125	120	46270
Insertions	5	2	1	2403
Deletions	43	23	28	5187
Positive Gaps	2679	2471	2925	26495
Internal Gaps	0	0	0	21
External Gaps	2679	2471	2925	26474
- total length	547408	558921	589979	19064103
- average length	204	226	202	720
Negative Gaps	110888	218908	110811	18198
Internal Overlaps	0	0	0	17
External Overlaps	110888	218908	110811	18181
- total length	−10247647	−20078971	−9424823	−1859835
- average length	−92	−92	−85	−102
Redundant Contigs	864	1158	607	6329
Unaligned Contigs	3262	4686	3221	60563
- partial	18	57	21	3252
- total length	462668	599320	447922	27666823
Ambiguous Contigs	2631	3876	2619	799
- total length	369284	483895	366418	93102

Metrics of the assemblies of the dataset c22 as delivered by Readjoiner (RJ), SGA, Edena and LEAP (*ℓ*_*min*_=45). The metrics are explained in [26] and in [28].

### Effect of sequencing errors

*Readjoiner* is based on the computation of exact suffix-prefix matches. Real-world datasets, however, contain a certain amount of errors. To better assess the effect of sequencing errors on the assembly, we sampled two sets of reads from the *Escherichia coli* K-12 genome, each consisting of 2 million reads: The first one (*Ecoli-without-errors*) is error-free, while in the second read set (*Ecoli-with-errors*) sequencing errors were introduced using a 0.75% position-independent substitution rate.

In order to assess the efficiency of *k*-mer based error correction (which we plan to implement in *Readjoiner*), we pre-processed *Ecoli-with-errors* using SGA. This constructs an FM-index from the reads set and errors are corrected using sga correct, delivering the read set termed *Ecoli-SGA-corrected*. This is further processed by sga filter, after constructing a new FM-index, to eliminate further error-containing reads. This resulting set *Ecoli-SGA-corrected+filtered* was assembled using both *Readjoiner* and SGA. Table 5 gives the most important statistics of the *Readjoiner* and SGA assemblies of the four different read sets defined here.

**Table 5:** Assembly of error-containing reads

	N50 (bp)		NG50 (bp)	
	RJ	SGA	RJ	SGA
*Ecoli-without-errors*	54948	54936	57213	57210
*Ecoli-with-errors*	203	5110	245	8645
*Ecoli-SGA-corrected*	38178	40002	39999	40824
*Ecoli-SGA-corrected+filtered*	41872	41872	41905	41903

N50 and NG50 values for the Readjoiner and SGA assemblies of Ecoli-without-errors, Ecoli-with-errors, Ecoli-SGA-corrected and Ecoli-SGA-corrected+filtered.

## Discussion and conclusion

In this paper, we presented methods and implementation techniques of a new string graph based assembler, named *Readjoiner*, which is significantly faster or more space efficient than the previous software tools Edena [9], SGA [11] and LEAP [13]. In particular, *Readjoiner* can handle a set of reads with 40× coverage of the entire human genome (total length of all reads 115 Gbp) on a machine with 64 GB RAM.

Although the different string graph-based assemblers aim at constructing the same graph, they apply different heuristics to compute a layout from the string graph. The quality of assemblies of simulated datasets was compared using metrics from the Plantagora project [26] and the Assemblathon 1 project [28]. In the assemblies of c22 delivered by *Readjoiner*, SGA and Edena there are 4 misassembled contigs. In contrast, 53.4% of the contigs of the LEAP assembly could not be aligned to the reference and 4.3% of the aligned contigs were misassembled. The “Negative Gaps” metric computed by Plantagora reflects the amount of overlaps among the contigs. Its high value for all tools can be explained by the fact that branching nodes in the string graph start new contigs in which the read corresponding to the branching node is included. Additionally considering the “Positive Gaps” metrics, one can conclude that most contigs were interrupted due to the presence of repetitive sequences, but not due to low coverage.

Our main development is a new efficient algorithm to compute all irreducible suffix-prefix matches from which the string graph is constructed. While the basic techniques we use (e.g. integer encodings, suffix sorting, integer sorting, binary search, bottom-up traversal of lcp-interval trees) are mostly well-established in sequence processing, their combination is novel for the considered problem. The different techniques were chosen with the overall goal of performing as few as possible random accesses to large data structures to obtain algorithms with excellent data locality which in turn leads to short run times. For most parts of our method, this goal was achieved, mostly due to the partitioning of the set of SPM-relevant suffixes. There are still many random accesses to the representation of the reads, which, however, cannot fully be prevented in an index based approach.

The problem of computing suffix-prefix matches has long been studied in the literature, mostly with the goal of finding, for each pair of reads *r* and *s*, the longest suffix-prefix match of *r* and *s*. Gusfield et al. [29] solved this maximum suffix-prefix matching problem in optimal *O*(*n* + *m*^2^) time and optimal *O*(*n*) space using the suffix tree for all suffixes of *m* reads of total length *n*.

Ohlebusch and Gog [30] present a solution to the same problem with the same time and space complexity using a linear scan of an enhanced suffix array. We do not know of any solution of the maximum suffix-prefix match problem which appropriately handles the reverse complements of the reads. Applying the algorithms of [29] or of [30] to the set of all reads and their reverse complements would not guarantee the maximality constraint, as the forward and reverse complement of a read are represented in different locations of the employed index structure.

In Edena, suffix-prefix matches are computed using a suffix array. Details of the algorithm or the implementation are not published.

Like Simpson and Durbin [11], we replaced the maximality constraint by a minimum length constraint imposed on each suffix-prefix match. The modified problem allows for an algorithm with two important advantages (compared to the algorithms of [29,30]): At first, the algorithm does not require a stack for each of *m* reads, and still can employ the space and time efficient bottom-up traversal of an lcp-interval tree as presented in Algorithm 1. Moreover, the algorithm can easily handle reverse complements of the reads and efficient selection of irreducible suffix-prefix matches is possible.

There are two main approaches to the construction of a string graph. The original approach of Myers [8] was to first construct a full overlap graph before transitive edges are removed. The resulting string graph contains all information relevant for the layout of the contigs. As the string graph contains much less edges than the overlap graph (the ratio depends on the coverage of the read set, see [8]), the explicit representation of this usually defines the space peak.

An alternative overlap graph representation for exact suffix-prefix matches was introduced in [13] and implemented in the LEAP software. The basic idea of this approach is to implicitly store many suffix-prefix matches for a set of lexicographically related reads in constant space using an interval representation. This allows for a compact storage of the full overlap graph. The representation does not apply to irreducible *SPM*s. In [13] only asymptotic results regarding the space requirement of the compact overlap graph representation are given, and LEAP does not give any clues on the size of the graph it constructs. So it remains unclear if this representation of the overlap graph is smaller than our representation of the string graph. A comparison of the overall space requirement of LEAP and *Readjoiner* shows a clear advantage for *Readjoiner*, see Table 3 for details.

It is worthwhile to note that the contigs output by LEAP contain many differences with respect to the target sequences they were sampled from. It is not clear to us, whether this is an artifact of the method or an implementation issue.

Another efficient way to reduce the space peak for string graph constructions is to recognize transitive *SPM*s and prevent their insertion in the graph structure. Simpson and Durbin [11] developed the first method following this approach and implemented it in the SGA software. In this paper, we have described an alternative algorithm, exploiting a property of transitive *SPM*s that can easily be checked on a small set of strings.

Our comparative tests (Table 2) indicate that *Readjoiner* is more than one order of magnitude faster than the current SGA implementation and uses less space. This may come as a surprise as SGA uses a compact index structure based on the BWT, while *Readjoiner* employs techniques known from enhanced suffix arrays, which are usually more space consuming. The space advantage of *Readjoiner* is mainly a result of our partitioning approach applied to the array of SPM-relevant suffixes. The partitioning technique leads to a large reduction in the overall memory peak and a small increase in the running time. This can be explained by an improved cache coherence: For a given part, only a small portion of the different tables are accessed. This seems to outweigh the time for the additional passes over the reads.

We see two reasons for the time advantage of *Readjoiner*: at first it employs a suffix selection and sorting method which is specifically tailored for the suffix-prefix matching problem and the given minimum match length ℓ_*min*_. In contrast, the BWT employed by SGA provides a general string indexing technique that is not optimized for computing *SPM*s of an arbitrary but fixed minimum length. Secondly, *Readjoiner* computes suffix-prefix matches by a linear scan of two integer tables, which is a very fast operation. In contrast, SGA relies on random accesses to the BWT which may take longer for large data sets.

The minimum match length parameter ℓ_*min*_ is used to restrict the search to the exact *SPM*s that are considered to be significant. To balance the required computational resources and the quality of the assembly, one has to carefully choose an appropriate value for ℓ_*min*_ = 45. A larger value of ℓ_*min*_ reduces the number of SPM-relevant suffixes, and in turn speeds up the computation and reduces the space requirement, but may lead to a poor assembly. Interestingly, in our simulations based on reads of length 100 bp, we obtained the best assembly results for a relatively large value of ℓ_*min*_ around 65. However, for a fair benchmarking of the tools and to simplify comparison with previous publications, we have chosen ℓ_*min*_ = 45.

Among the string graph-based assemblers mentioned here, SGA is the only one that can distribute parts of the computation across multiple threads. Some of the algorithms employed in *Readjoiner* are well suited for a multi-threaded implementation. For example, each bucket of SPM-relevant suffixes is sorted independently and the corresponding *SPM*s are computed independently of all other buckets. This step only requires random read access to the representation of the reads. A multi-threaded implementation with shared memory access to the reads and buckets which are (with respect to their sizes) evenly distributed over the threads, is expected to provide a considerable speedup within a small amount of additional space.

Another important issue for future development is the improvement of the assembly quality for real world data. Here further preprocessing steps, in particular quality filtering and error detection are required, as well as the handling of paired read information in the assembly phase.

The present manuscript focuses on the algorithmic approach and implementation of methods for the computation of irreducible suffix-prefix matches and the construction of the string graph. We report our results on error-free datasets: This is in analogy to the first papers describing the methods implemented in SGA [11] and LEAP [13].

Several error correction strategies have been applied so far: The classical method was to consider approximative suffix-prefix matches of the reads and to correct the resulting contigs in a consensus phase. With large next-generation datasets, the method of choice consist in *k*-mer counting, identification of a subset of trusted *k*-mer, which occur at least a given number of times in the read set, and correction of the reads containing untrusted *k*-mers [12,31].

Approximative suffix-prefix matching algorithms can be implemented to work on index structures, but the increased search space makes them significantly slower than exact matching algorithms. Among the string graph-based assemblers, only SGA implements an approximate suffix-prefix matching algorithm: Nevertheless, this is not used by default, and the authors recommend using their faster *k*-mer based error correction method instead [12].

The fact that *Readjoiner* is based on exact suffix-prefix matches makes it sensible to errors. We have demonstrated that using a *k*-mer based error correction step delivers read sets for which *Readjoiner* delivers assemblies with metrics comparable to SGA. We therefore plan to implement a *k*-mer based error correction for *Readjoiner*, employing techniques similar to those used for computing suffix-prefix matches.

Paired-end and mate pairs provide short and long range positional information, which is critical for improving the quality of assembling eukaryotic genomes. The classical approach consists in using this information for connecting contigs into scaffolds either in a post-processing phase, which may be integrated in the assembler software, or using a stand-alone tool, such as Bambus [32] or SSPACE [33]. A complementary approach, which we intend to introduce in a future version of our software, is to exploit the pairing information already during the traversal of the string graph, by restricting to paths connecting the mate pairs with a length compatible to the insert size of the library. Details of such an approach are given in [34,35].

## Availability

The *Readjoiner* software is available as part of the *GenomeTools* genome analysis package [21], a free, open source collection of bioinformatics software. See http://www.zbh.uni-hamburg.de/readjoiner for more details.

## Authors’ contributions

GG developed most of the methods, implemented *Readjoiner*, and performed the benchmarks. SK conceived the project and developed the suffix selection and sorting methods. Both authors wrote the manuscript and approved its final version.

## Supplementary Material

Additional file 1 **Supplemental Material.** This document describes implementation techniques for the methods and algorithms described in the main document. Moreover, it gives a lemma and a theorem (including proofs) characterizing transitive SPMs, and an algorithm to enumerate irreducible and non-redundant suffix-prefix matches. Furthermore, a method to recognize internally contained reads is given, as well as results for a benchmark set with reads of variable length. Finally, an example of SPM-relevant suffixes and their corresponding lcp-interval is presented. Click here for file
