# Supplementary material for: Readjoiner: a fast and memory efficient string graph-based sequence assembler
Source: BMC Bioinformatics. 2012 May 6;13:82. doi: 10.1186/1471-2105-13-82 (PMC3507659; doi:10.1186/1471-2105-13-82)
Supplement: Additional file 1 — Supplemental Material. This document describes implementation techniques for the methods and algorithms described in the main document. Moreover, it gives a lemma and a theorem (including proofs) characterizing transitive SPMs, and an algorithm to enumerate irreducible and non-redundant suffix-prefix matches. Furthermore, a method to recognize internally contained reads is given, as well as results for a benchmark set with reads of variable length. Finally, an example of SPM-relevant suffixes and their corresponding lcp-interval is presented. [file 1471-2105-13-82-S1.pdf]

Additional file 1 for manuscript Readjoiner: a fast and memory efficient string graph-based sequence assembler

Giorgio Gonnella   Stefan Kurtz
Center for Bioinformatics
University of Hamburg

March 1, 2012

This document describes implementation techniques for the methods and algorithms described in the main document. Moreover, it gives a lemma and a theorem (including proofs) characterizing transitive SPMs, and an algorithm to enumerate irreducible and non-redundant suffix-prefix matches. Furthermore, a method to recognize internally contained reads is given, as well as results for a benchmark set with reads of variable length. Finally, an example of SPM-relevant suffixes and their corresponding lcp-interval is presented.

1 Implementation of counts

In this section we describe how to efficiently implement the counters. Recall that we have d counters, one for each initial k-mer. The sum of all counts is exactly the number g of all SPM-relevant suffixes. This implies that the expected value of a counter is $\frac{g}{d}$. As $g \leq n$ and $d \geq m$, we have $\frac{g}{d} \leq \frac{n}{d} \leq \frac{n}{m}$. As the average read length $\frac{n}{m}$ is assumed to be a constant, the expected value of a counter is a constant as well and so we implement the count table C by a byte array of size d. The counts stored in this array are in the range from 1 to 255, as each count refers to an integer code that occurs at least once. So 0 is a special value. If the ith integer code occurs more than 255 times, we set the ith entry of the byte array to 0, to signify an overflow and store the correct count with key i in a hash table. Because we use sorted buffers in the counting phase, the array-indices of the counters to be incremented come

in sorted order. So in each round q consecutive increments by 1 (and this many accesses to the byte array and possibly to the hash table) can be combined into one increment by q. Thus, for each round, each counter is incremented at most once. This strategy reduces the number of accesses to the hash table. For the datasets of the results section only about 0.2% of all counters have to be stored in the hash table and less than 1% of all increments affect the hash table. Thus the effect of the hash table on the space and the running time is negligible.

2 Implementation of partial sums

The partial sums in table π are bounded by g, the number of SPM-relevant suffixes. For large read sets, g can be larger than $2^{32}-1$. However, as the partial sum are strictly increasing, one can implement π by a 32 bit integer table PS of size $d+1$, such that $\mathsf{PS}[i] = \pi[i] \bmod 2^{32}$ for any i, $0 \leq i \leq d$ and an additional integer table of size $2^{\max\{0,\lceil\log_2 g\rceil-32\}}$. Let $\beta = \lceil\log_2 g\rceil$ and δ be some small constant such that each count value in table C is smaller than 2^δ. Each value $\pi[i]$ is in the range from 0 to g and it can be written as

$$\pi[i] = \pi[i] \bmod 2^\delta + \sum_{j=1}^{\beta-\delta} b_j 2^{\beta-j} \tag{1}$$

where $b_j \in \{0,1\}$ for all j, $1 \leq j \leq \beta-\delta$. So in particular, if $\pi[i] < 2^\delta$ then $\pi[i] = \pi[i] \bmod 2^\delta$ and so the bits $b_1,\dots,b_{\beta-\delta}$ are all 0. As the sequence of partial sums is strictly increasing, we store for each possible combination of bits $b_1,\dots,b_{\beta-\delta}$ the largest i, $0 \leq i \leq d$ satisfying (1). This value i is stored at index $b_1 \dots b_{\beta-\delta}$ of a table f of size $2^{\beta-\delta}$, where the bit sequence $b_1 \dots b_{\beta-\delta}$ is interpreted as a decimal number. In other words, we store the least significant δ bits of $\pi[i]$ in $\mathsf{PS}[i]$ and the most significant $\beta-\delta$ bits are interpreted as an index into table f.

As the partial sums are computed sequentially in one pass over the count table C, it is easy to fill table f. When retrieving $\pi[i]$ one first determines the smallest q such that $i \leq f[q]$ for some q, $0 \leq q \leq 2^{\beta-\delta}-1$ and returns $\mathsf{PS}[i] + q \cdot 2^\delta$. As $\beta-\delta$ is a very small constant, q and this $\pi[i]$ can be determined in constant time. δ can be chose such that that the sum of the sizes of table PS and f is minimized. However, in our implementation we have chosen $\delta = 32$.

3 Implementation of the partitioning strategy

In our implementation of the partitioning strategy we exploit the fact that the size and content of the tables K and P do not change during the insertion phase, so that they can conveniently be stored on temporary files after the partial sums have been computed and before the insertion phase begins. As we do not want to afford the space for the entire table PS, we directly output the sequence of partial sums to a temporary file when they are generated. We only store a small sample of the partial sums in memory, allowing to determine the boundaries at which the different parts of the tables are partitioned. Using a small sample of π instead of π completely leads to a more granular partitioning and thus to a larger variance of the size of the different parts. However, our experiments show that the variance is still very small.

When processing the q parts, one after the other, the range of values of table K and P (only read access) and PS (read and write access) in the predefined boundaries are mapped from the temporary files into main memory in units of the machine's page size. The access to the temporary files is strictly sequential and only the values on pages at the boundaries of the parts may be mapped more than once. As a consequence, our partitioning strategy only creates a minimal overhead in the IO subsystem of the computer.

4 Computation of leaf edges in Algorithm 1

The additional computations in lines 6–12 and line 32 of Algorithm 1 (see main document) deliver the leaf edges. These were omitted in the original algorithm of [1, Algorithm 4.4]. To understand how the computation works, first note that at the end of each iteration of the for-loop, $stack.top.lcp$ equals L_{e+1} and $lastitv$ is undefined. The singleton-interval $[e]$ representing H_e has a parent with lcp-value $\ell = \max\{L_e, L_{e+1}\}$. So at the start of the eth iteration we have $stack.top.lcp = L_e$. Hence, if $L_{e+1} \leq stack.top.lcp$, then the parent of $[e]$ has lcp-value $\ell = stack.top.lcp$. Since $stack.top.lb \leq e$ and $stack.top.rb$ will get a value $\geq e$ in later iterations, $stack.top$ represents the parent of $[e]$. This case is implemented in lines 6–12.

Now suppose that $L_{e+1} > stack.top.lcp$. Then the while-loop is not executed and $lastitv$ remains undefined. So the next statement executed is in line 31. $L_{e+1} > stack.top.lcp$ implies $L_{e+1} > L_e$ and so the parent of $[e]$ has the lcp-value L_{e+1}. This parent $(L_{e+1}, e, \perp)$ is pushed on the stack in line 31 and the implicit outgoing leaf edge is processed in line 32.

What remains is an explanation on how to determine if an edge is the first outgoing from an lcp-interval. As the edges processed in line 28 or line 32 are from lcp-intervals which have just been generated (in the line before), these edges are obviously the first edges from the lcp-interval (with undefined right bound). Now look at the identical code fragment in lines 7–11 and 18–22. If the current lcp-interval is not the root-interval (of lcp-value 0), then there was a previous edge outgoing from this lcp-interval (processed either in line 28 or 32) and so $firstedge$ is false. Otherwise, if the current lcp-interval is the root-interval, then $firstedge$ is true if and only if $firstedgefromroot$ is true. The latter boolean variable is true if and only if there was some previously processed edge from the root-interval. $firstedgefromroot$ is set to false whenever an edge from the root-interval is processed.

5 Computation of integer codes for reverse complemented reads

The three steps which involve scanning the reads are extended to process both strands of all reads. This does not require to double the size of the read representation, as all information for the reverse complemented reads can efficiently be extracted from the forward reads. Here we show how to compute the integer codes for the reversed reads from the integer codes of the forward reads in constant time. More precisely, the computation of $c_{rev} := \varphi_k(s[k]s[k-1]\ldots s[1])$ from $c := \varphi_k(s[1\ldots k])$ can be done with $3k-1$ bit operations if k is even and $3k-2$ bit operations if k is odd. These bit operations reverse the order of k pairs of bits. For

computing the complement of a k-mer, first note that the following equations hold (where xor denotes bitwise exclusive or):

$$\begin{aligned}\varphi(A) = 0 \text{ xor } 3 = 3 = \varphi(T)\\ \varphi(C) = 1 \text{ xor } 3 = 2 = \varphi(G)\\ \varphi(G) = 2 \text{ xor } 3 = 1 = \varphi(C)\\ \varphi(T) = 3 \text{ xor } 3 = 0 = \varphi(A)\end{aligned}$$

That is, $(\text{xor } 3)$ applied to the code of a base delivers the code of the complement of the base. Of course, this works for a bit string with an even number of bits, so that for a k-mer u of $2k$ bits, $\varphi_k(u)$ xor 3 delivers the integer code of the complement of u. Thus applying this operation to c_{rev} gives the integer code of the reverse complement of $s[1 \ldots k]$ in constant time. The window-based approach described in the implementation section can be extended to simultaneously compute the integer code for the reverse complement of the k-mer in the window.

6 Characterization of transitive suffix-prefix matches

The following lemma characterizes an *SPM* by a read and a single *SPM* satisfying a length constraint and a match constraint.

Lemma 1 Let $\langle r, t, \ell''\rangle$ be an *SPM*. Then $\langle r, t, \ell''\rangle$ is transitive, if and only if there is an $s \in \mathcal{R}$ and an *SPM* $\langle s, t, \ell'\rangle$ such that $\ell' > \ell''$, $|r| - \ell'' \geq |s| - \ell'$ and $s[1 \ldots |s| - \ell'] = r[|r| - \ell'' - (|s| - \ell') + 1 \ldots |r| - \ell'']$.

Proof:

"⇒": Suppose that $\langle r, t, \ell''\rangle$ is transitive. Then, by definition, there is an $s \in \mathcal{R}$ and two *SPM*s $\langle r, s, \ell\rangle$ and $\langle s, t, \ell'\rangle$ such that $\ell+\ell' > |s|$. We have to show that $\ell' > \ell''$, $|r|-\ell'' \geq |s|-\ell'$ and $s[1 \ldots |s| - \ell'] = r[|r| - \ell'' - (|s| - \ell') + 1 \ldots |r| - \ell'']$. Because s is not a prefix of t, $\ell' < |s|$. By definition, there are sequences u, v, w, x, and y such that $r = uvw$, $s = vwx$, $t = wxy$, $\ell = |vw|$, and $\ell' = |wx|$. This implies

$$|w| = |v|+|w|+|w|+|x|-(|v|+|w|+|x|) = |vw|+|wx|-|vwx| = \ell+\ell'-|s| > 0.$$

Hence w is a non-empty suffix of r and a non-empty prefix of t which implies that $\langle r, t, \ell''\rangle$ is an *SPM* of length $\ell'' = \ell + \ell' - |s|$. As $\ell' = |wx|$ and $\ell'' = |w|$, we conclude $\ell' \geq \ell''$. Assume that $\ell' = \ell''$. Then $x = \varepsilon$ which implies that $s = vw$ is a suffix of r. This contradicts the fact that $\mathcal{R}$ is suffix-free. Thus our assumption was wrong, which implies $\ell' > \ell''$. Since $s = vwx$, we obtain $|v| = |s|-\ell'$ and $|r| \geq |v|+|w| = |s|-\ell'+\ell''$ from which we conclude $|r| - \ell'' \geq |s| - \ell'$. Moreover, from $s = vwx$ and $r = uvw$, we derive $s[1 \ldots |s| - \ell'] = v = r[|r| - \ell'' - (|s| - \ell') + 1 \ldots |r| - \ell'']$.

"⇐": Suppose there is an $s \in \mathcal{R}$ and an *SPM* $\langle s, t, \ell'\rangle$ such that $\ell' > \ell''$, $|r| - \ell'' \geq |s| - \ell'$ and $s[1 \ldots |s| - \ell'] = r[|r| - \ell'' - (|s| - \ell') + 1 \ldots |r| - \ell'']$. We have to show that $\langle r, t, \ell''\rangle$ is

transitive. Let w, x be strings such that wx of length $\ell' > 0$ is a suffix of s and a prefix of t and w of length $\ell'' > 0$ is a suffix of r and a prefix of t. Let $v = s[1 \ldots |s| - \ell']$. Then, by assumption, $v = r[|r| - \ell'' - (|s| - \ell') + 1 \ldots |r| - \ell'']$ and thus vw is a suffix of r and a prefix of s. We have $|vw| = |s| - \ell' + \ell'' > 0$ and thus there is an *SPM* $\langle r, s, \ell \rangle$ where $\ell = |s| - \ell' + \ell''$. Since $\ell' = |wx|$ and $\ell = |vw|$, we conclude $\ell + \ell' = |w| + |x| + |v| + |w| = |w| + |vwx| = |w| + |s| > |s|$. So $\langle r, t, \ell'' \rangle$ is transitive.

□

There is an even more stringent characterization of transitive *SPM*s based on a single irreducible *SPM*, as stated in the following theorem.

Theorem 1 Let $\langle r, t, \ell'' \rangle$ be an *SPM*. Then $\langle r, t, \ell'' \rangle$ is transitive if and only if there is an $s \in \mathcal{R}$ and an irreducible *SPM* $\langle s, t, \ell' \rangle$ such that $\ell' > \ell''$, $|r| - \ell'' \geq |s| - \ell'$ and $s[1 \ldots |s| - \ell'] = r[|r| - \ell'' - (|s| - \ell') + 1 \ldots |r| - \ell'']$.

Proof:

"⇒": Let $\langle r, t, \ell'' \rangle$ be transitive. Then, by Lemma 1, there is an $s \in \mathcal{R}$ and an *SPM* $\langle s, t, \ell' \rangle$ such that $\ell' > \ell''$, $|r| - \ell'' \geq |s| - \ell'$ and $s[1 \ldots |s| - \ell'] = r[|r| - \ell'' - (|s| - \ell') + 1 \ldots |r| - \ell'']$. Without loss of generality we assume that ℓ' is maximal, i.e. for any $s' \in \mathcal{R}$ and any *SPM* $\langle s', t, q \rangle$ such that $q > \ell''$, $|r| - \ell'' \geq |s'| - q$ and $s'[1 \ldots |s'| - q] = r[|r| - \ell'' - (|s'| - q) + 1 \ldots |r| - \ell'']$ holds, we have $q \leq \ell'$. Now assume that $\langle s, t, \ell' \rangle$ is transitive. Then by Lemma 1, there is an $s' \in \mathcal{R}$ and an *SPM* $\langle s', t, q \rangle$ such that $q > \ell'$, $|s| - \ell' \geq |s'| - q$ and $s'[1 \ldots |s'| - q] = s[|s| - \ell' - (|s'| - q) + 1 \ldots |s| - \ell']$. First note that $q > \ell'$ and $\ell' > \ell''$ implies $q > \ell''$. Moreover, $|r| - \ell'' \geq |s| - \ell' \geq |s'| - q$. This implies $|r| - \ell'' - (|s'| - q) + 1 \geq 1$, i.e. $r[|r| - \ell'' - (|s'| - q) + 1 \ldots |r| - \ell'']$ is defined. From the two applications of Lemma 1 we conclude

$$\begin{aligned} s'[1 \ldots |s'| - q] &= s[|s| - \ell' - (|s'| - q) + 1 \ldots |s| - \ell'] \\ &= r[|r| - \ell'' - (|s| - \ell') + 1 + |s| - \ell' - (|s'| - q) \ldots |r| - \ell''] \\ &= r[|r| - \ell'' - |s| + \ell' + 1 + |s| - \ell' - (|s'| - q) \ldots |r| - \ell''] \\ &= r[|r| - \ell'' - (|s'| - q) + 1 \ldots |r| - \ell'']. \end{aligned}$$

This implies $q \leq \ell'$, which is a contradiction. Hence our assumption that $\langle s, t, \ell' \rangle$ is transitive was wrong. In other words, $\langle s, t, \ell' \rangle$ is irreducible, which was to be shown.

"⇐": Suppose there is an $s \in \mathcal{R}$ and an irreducible *SPM* $\langle s, t, \ell' \rangle$ such that $\ell' > \ell''$, $|r| - \ell'' \geq |s| - \ell'$ and $s[1 \ldots |s| - \ell'] = r[|r| - \ell'' - (|s| - \ell') + 1 \ldots |r| - \ell'']$. Then by Lemma 1, $\langle r, t, \ell'' \rangle$ is transitive.

□

7 Computation of non-redundant irreducible suffix-prefix matches

Algorithm 3 is a modification of Algorithm 2 (see main document) to output non-redundant irreducible *SPM*s only. The set W now contains pairs (j, D_j), where j refers to the read with number j and D_j is the dictionary of left contexts of suffixes corresponding to terminal edges on the whole-read path for r_j. Whenever, on the whole-path for read r_j, a terminal edge outgoing from an lcp-interval itv and starting with $\$_p$ is detected, $LCsearch(\mathsf{D}_j, \mathsf{LC}(r_p, itv.lcp))$ is called. The *SPM* $\langle r_p, r_j, itv.lcp\rangle$ is output if and only if the function call returns false.

A dictionary is removed once an lcp-interval itv with $itv.lcp < \ell_{min}$ is visited. This makes sense, as in this case no more *SPM*s $\langle \_, r_p, \ell\rangle$ satisfying $\ell \geq \ell_{min}$ will be found.

$LCsearch(\mathsf{D}, s)$ requires $O(|s|)$ time. As the length of s is bounded by a constant, the running time of $LCsearch(\mathsf{D}, s)$ is constant. Hence, the total length of Algorithm 3 is $O(n+z)$ where z is the number of processed *SPM*s.

8 Recognition of internally contained reads

The following lemma characterizes internally contained reads in terms of an lcp-interval tree.

Lemma 2 Let $\mathcal{R}$ be a suffix- and prefix-free read set with reads of length at least ℓ_{min}. Let T be the lcp-interval tree of the SPM-relevant suffixes of all reads in $\mathcal{R}$. For any read $r \in \mathcal{R}$, r is internally contained if and only if T contains an r-interval with exactly one terminal edge to a singleton whole-read interval representing r.

Proof:

"$\Rightarrow$": Suppose that r is internally contained. Then there is some read r' such that $r' = urv$ for some non-empty strings u and v. As the first base of v is different from the sentinel with which r is padded, there is an r-interval $[e..f]$ with an outgoing terminal edge to a singleton whole-read interval (representing r). Suppose $[e..f]$ has another outgoing terminal edge to a singleton whole-read interval (representing some read r''). Then $r = r''$, which contradicts the assumption that $\mathcal{R}$ is prefix- and suffix-free. Hence in T there is exactly one terminal edge outgoing from $[e..f]$ and leading to a singleton whole-read interval.

"$\Leftarrow$": Suppose that in T there is an r-interval $[e..f]$, with exactly one terminal edge to a singleton whole-read interval representing read r. By construction $[e..f]$ has another outgoing edge whose label v does not start with a sentinel. Suppose the rv is a prefix of some read. Then the path from $[e..f]$ via the edge label v must lead to a singleton whole-read interval. Hence rvv' is a read for some string v'. Hence r is a prefix of a longer read, which contradicts the fact that $\mathcal{R}$ is prefix-free. Hence rv is not a prefix of some read, but rvv' is a proper suffix of some read. Hence there is some non-empty string u such that $urvv'$ is a read. Hence r is internally contained in $urvv'$.

□

Algorithm 3 Bottom-up computation of all non-redundant irreducible suffix-prefix matches in $\overline{\mathcal{R}}$.

Input: table L and sorted array H of SPM-relevant suffixes of $\overline{\mathcal{R}}$ with common prefix u of length k

Output: Non-redundant irreducible suffix-prefix matches $\langle r, s, \ell \rangle$ such that $\ell \geq \ell_{min}$, $r, s \in \overline{\mathcal{R}}$, u is a prefix of s

$T := [\,]$ ▷ empty list
$W := [\,]$ ▷ empty list of pairs (i, D_i), $1 \leq i \leq m$, D_i is a left-context dictionary
with each lcp-interval itv associate an integer $itv.firstinW$
run Algorithm 1 with the following functions:
function $process\_leafedge(firstedge,\ itv,\ (p, q))$ ▷ p is read number and q is offset
  if $itv.lcp \geq \ell_{min}$ **then**
    if $firstedge$ **then**
      $itv.firstinW := |W| + 1$
    if $q = 0$ **then** ▷ (p, q) refers to whole read
      create a new empty dictionary D_p
      append (p, D_p) to W
    if $q + itv.lcp = |r_p|$ **then** ▷ (p, q) refers to terminal edge
      append p to T
  else
    $W := [\,]$
function $process\_branchedge(firstedge, itv, itv')$
  if $itv.lcp \geq \ell_{min}$ **then**
    if $firstedge$ **then**
      $itv.firstinW := itv'.firstinW$
  else
    W := [ ]
function $process\_lcpinterval(itv)$
  if $itv.lcp \geq \ell_{min}$ **then**
    for all $p \in T$ **do**
      for all $(j, \mathsf{D}_j) \in W[itv.firstinW \ldots |W|]$ **do**
        if $LCsearch(\mathsf{D}_j, \mathsf{LC}(r_p, itv.lcp)) = \mathsf{false}$ **then**
          $(d_p, p') :=$ **if** $p \leq m$ **then** (fwd, p) **else** $(\mathsf{rev}, p - m)$
          $(d_j, j') :=$ **if** $j \leq m$ **then** (fwd, j) **else** $(\mathsf{rev}, j - m)$
          if $d_p = \mathsf{fwd}$ **and** $p' \leq j'$ **or** $(d_p, d_j) = (\mathsf{rev}, \mathsf{fwd})$ **and** $p' \geq j'$ **then** ▷ non-redundant?
            output $\langle r_p, r_j, itv.lcp \rangle$
  T := [ ]

Algorithm 4 exploits Lemma 2 to detect internally contained reads. After applying the algorithm to all buckets of SPM-relevant suffixes, IC is the set of reads in $\mathcal{R}$ which are internally contained.

Algorithm 4 Bottom-up determination of internally contained reads

Input: table L and sorted array H of SPM-relevant suffixes in the ith bucket with common prefix u of length k

Output: Set IC of reads r_i which have prefix u and are internally contained.

1: $\mathsf{IC} := \emptyset$

2: run Algorithm 1 of main document with the following function ($process\_branchedge$ and $process\_lcpinterval$ are not needed):

3: **function** $process\_leafedge(firstedge,\ itv,\ (p,q))$ $\triangleright$ p is read number and q is read offset

4: **if** $q + itv.lcp = |r_p|$ **and** $q = 0$ **then**

5: $\mathsf{IC} := \mathsf{IC} \cup \{r_p\}$

Note that the conditions checked in line 4 of Algorithm 4 are the same as in line 9 and 11 of Algorithm 2 given in the main document. This allows to conveniently merge the two algorithms, in order to compute suffix-prefix matches and recognize internally contained reads by scanning the index structure only once.

9 Performance on variable length datasets

The results presented in the main document refer to read sets in which each read has the same length (100 bp). This situation is common for datasets obtained by the Illumina sequencing platforms. Read in datasets from asynchronous sequencing technologies, such as Roche 454, vary in their lengths. Here we present a benchmark on a read set with reads of variable length.

Dataset c22_454 was generated taking human chromosome 22 as a template, with $20\times$ coverage and read lengths randomly sampled from the distribution of read lengths of a real-world 454 dataset (obtained from the Short Read Archive, accession DRR000841).

The reads were assembled using *Readjoiner*, LEAP and SGA. Results are shown Table 6. Edena was excluded from this benchmark as is requires reads of equal length.

Table 6: Results of applying *Readjoiner* (RJ), SGA and LEAP to the dataset c22_454. ($\ell_{min} = 45$). The readset consists of 2.0 million reads with a total length of 697.9 Mbp. Read length is variable, between 26 bp and 637 bp.

Benchmark results			
	RJ	SGA	LEAP
Running time (s)	477	6324	1000
Space peak (Mb)	245	343	550

Assemblathon metrics			
	RJ	SGA	LEAP
Number of contigs	16572	26158	26204
Genome size (bp.)	34894545	34894545	34894545
Total contigs length	42073401	45137152	42348355
- as % of genome	120.57	129.35	121.36
Mean contig size	2538.82	1725.56	1616.10
Median contig size	907	588	781
Longest contig	73334	73334	47718
Shortest contig	395	92	250
Contigs $>$ 500 bp	16243 (98.01 %)	18438 (70.49 %)	22720 (86.70 %)
Contigs $>$ 1K bp	7353 (44.37 %)	6947 (26.56 %)	10730 (40.95 %)
Contigs $>$ 10K bp	830 (5.01 %)	793 (3.03 %)	343 (1.31 %)
N50	6155	5089	2786
L50	1694	2037	3644
NG50	8085	7674	3735
LG50	1182	1219	2471

Plantagora metrics			
	RJ	SGA	LEAP
Covered Bases	34789523	34781707	24513730
Ambiguous Bases	84743	81574	430827
Misassemblies	2	0	11551
Misassembled Contigs	1	0	6986
Misassembled Contigs Bases	836	0	15569182
SNPs	18	7	50856
Insertions	0	0	2584
Deletions	0	0	4842
Positive Gaps	203	231	3490
Internal Gaps	0	0	3
External Gaps	203	231	3487
- total length	109027	117608	5159684
- average length	537	509	1480
Negative Gaps	15936	24987	11853
Internal Overlaps	0	0	18
External Overlaps	15936	24987	11835
- total length	-6988125	-9831815	-6982471
- average length	-439	-393	-590
Redundant Contigs	74	160	12388
Unaligned Contigs	30	77	19
- partial	0	4	20
- total length	25141	52808	11833
Ambiguous Contigs	297	638	318
- total length	206437	350265	188769

10 An example of a set of SPM-relevant suffixes and the correspinding lcp-interval tree

Consider the readset $\mathcal{R} = \{aact, actt, ataa, aaca\}$. We have the following non-empty suffixes, of which the (ℓ_{min}, k)-SPM-relevant suffixes for $\ell_{min} = 2$ and $k = 1$ are underlined.

$$\begin{array}{rrrr} \underline{aact} & \underline{actt} & \underline{ataa} & \underline{aaca} \\ \underline{act} & ctt & taa & \underline{aca} \\ ct & tt & \underline{aa} & ca \\ t & t & a & a \end{array}$$

As all reads begin with a, there is only one bucket with all SPM-relevant suffixes. The following table shows the SPM-relevant suffixes in lexicographic order, including the lcp-values. Note that the sentinels are explicitly shown as they are relevant for the lexicographic order.

j	H_j as pair	H_j as string	L_j
0	$(4,1)$	$aaca\$_4$	
1	$(1,1)$	$aact\$_1$	3
2	$(3,3)$	$aa\$_3$	2
3	$(4,2)$	$aca\$_4$	1
4	$(2,1)$	$actt\$_2$	2
5	$(1,2)$	$act\$_1$	3
6	$(3,1)$	$ataa\$_3$	1

Here is the lcp-interval tree, represented by table S and L. The leaves are marked by the suffixes (in form of read number and read offset) they refer to. The notation ℓ-$[e..f]$ refers to the lcp-interval $[e..f]$ of lcp-value ℓ.

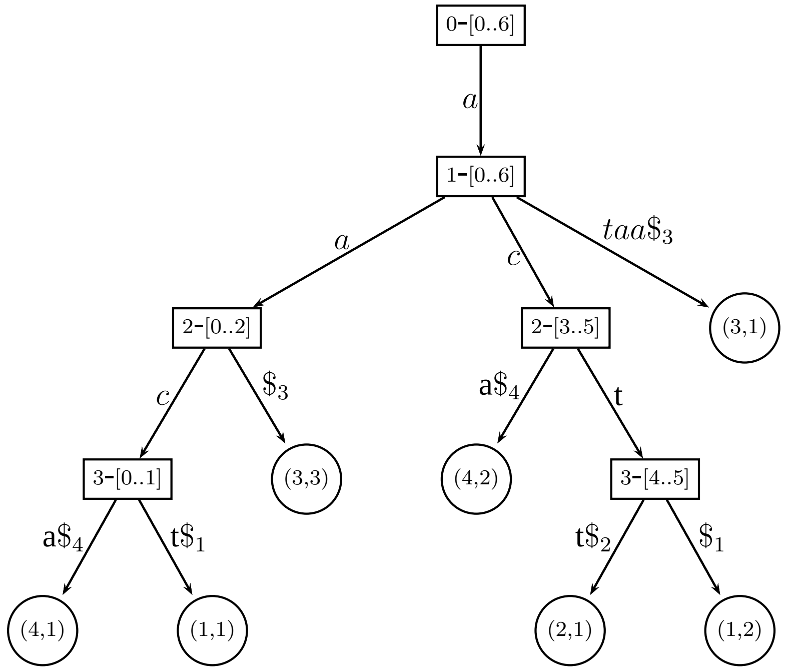

This is the list of lcp-intervals and singleton intervals, in the order in which they are processed:

$(4,1), (1,1), 3\text{-}[0..1], (3,3), 2\text{-}[0..2], (4,2), (2,1), (1,2), 3\text{-}[4..5], 2\text{-}[3..5], (3,1), 1\text{-}[0..6], 0\text{-}[0..6]$

References

[1] M.I. Abouelhoda, S. Kurtz, and E. Ohlebusch. Replacing suffix trees with enhanced suffix arrays. *Journal of Discrete Algorithms*, 2:53–86, 2004.
